# Metal Complexes of Singly, Doubly and Triply Linked Porphyrins and Corroles: An Insight into the Physicochemical Properties

**DOI:** 10.1002/chem.202104550

**Published:** 2022-03-07

**Authors:** Arijit Singha Hazari, Shubhadeep Chandra, Sanjib Kar, Biprajit Sarkar

**Affiliations:** ^1^ Lehrstuhl für Anorganische Koordinationschemie Institut für Anorganische Chemie Universität Stuttgart Pfaffenwaldring 55 70569 Stuttgart Germany; ^2^ School of Chemical Sciences National Institute of Science Education and Research (NISER) Bhubaneswar 752050 India; ^3^ Homi Bhabha National Institute Training School Complex Mumbai 400094 (India)

**Keywords:** fused porphyrins, directly linked corroles, metal complexes, electrochemistry, magnetic properties

## Abstract

Metal complexes of multi‐porphyrins and multi‐corroles are unique systems that display a host of extremely interesting properties. Availability of free *meso* and β positions allow formation of different types of directly linked bis‐porphyrins giving rise to intriguing optical and electronic properties. While the fields of metalloporphyrin and corroles monomer have seen exponential growth in the last decades, the chemistry of metal complexes of bis‐porphyrins and bis‐corroles remain rather underexplored. Therefore, the impact of covalent linkages on the optical, electronic, (spectro)electrochemical, magnetic and electrocatalytic activities of metal complexes of bis‐porphyrins and ‐corroles has been summarized in this review article. This article shows that despite the (still) somewhat difficult synthetic access to these molecules, their extremely exciting properties do make a strong case for pursuing research on these classes of compounds.

## Graphical abstract

**Metal complexes** of directly linked metalloporphyrine and corrole oligomers have been extensively studied due to their intriguing optical and electronic properties. In this regard, the impact of the different type of direct linkages on electrochemical, magnetic, electronic and electrocatalytic properties of various metallopophyrine and corrole oligomers have been discussed at length in this Review article.

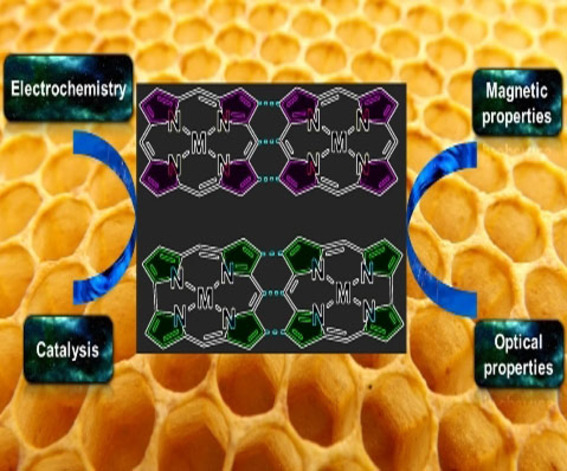

## 1 Introduction

Over the years, organic macrocycles with extended π‐conjugation have been extensively studied from the context of their possible applications in various research dimensions, namely molecular wires,^[1]^ organic semiconductor devices,^[2]^ NIR‐dyes,^[3]^ and non‐linear optical materials (NLO).^[4]^ Naturally, considerable attention has been devoted to the development of efficient strategies for their synthesis and characterization. Over the past three decades, research in the field of organic macrocycles brought about significant advancements regarding synthetic strategies and fundamental features of the molecules. However, these synthetic protocols often suffer from a serious setback dealing with low solubility and chemical instability, which become more prominent with a gradual increase in the number of macrocyclic units. Besides, one‐dimensional macrocycles with extended π‐conjugation have an inherent problem of saturation, defined by effective conjugation length (ECL).^[5]^ ECL signifies the optimum length of the π‐conjugated system up to which the electronic delocalization is limited, and the optical, electrochemical, and physicochemical properties tend to be saturated. Therefore, optimum solubility and stability are some of the desired properties for effective utilization of the conjugated macrocycles.

Tetrapyrrolic macrocycles are among the most studied type of organic macrocycles owing to their relevance in biological systems either in the metal‐free form^[6]^ or more commonly as metal complexes.^[6]^ Porphyrin, a unique class of organic heterocycles, comprises four pyrrole units connected by four methine carbon bridges in a coplanar fashion, thus creating a square planar metal coordination environment leaving both the axial positions unoccupied which consequently allows reversible binding and activation of various substrates. From the structural standpoint, it has an effective π‐conjugation across the entire framework attributing an aromatic behavior. The cavity inside the structure can, in principle, accommodate almost all the metals. The dianionic nature of the binding site provides necessary stabilization to high‐valent metal ions on the one hand, and on the other hand it also imparts strong reducing power to the low‐valent metal ions. Hence, metal complexes of porphyrins are of fundamental importance due to their involvement in various biological processes like O_2_ binding and transport (hemoglobin and myoglobin),^[7]^ activation of O_2_ and utilization (Cytochrome P450),^[8]^ and degradation and management of peroxide in enzymes (peroxidase and catalase).^[9]^ Additionally, metalloporphyrins have also found wide applications in numerous research fields like photodynamic therapy,^[10]^ catalysis,^[11]^ artificial photosynthesis,^[12]^ and sensors^[13]^ owing to their various advantageous properties like structural robustness, superior catalytic activity, promising absorption and emission, and rich coordination chemistry.^[14]^ Literature reports over the years have shown that physicochemical properties of porphyrins can be perturbed through substitution at the peripheral positions, which in turn provides a scope of rational fine‐tuning resulting in drastically altered optical and electronic properties.^[15]^

**Scheme 1:**
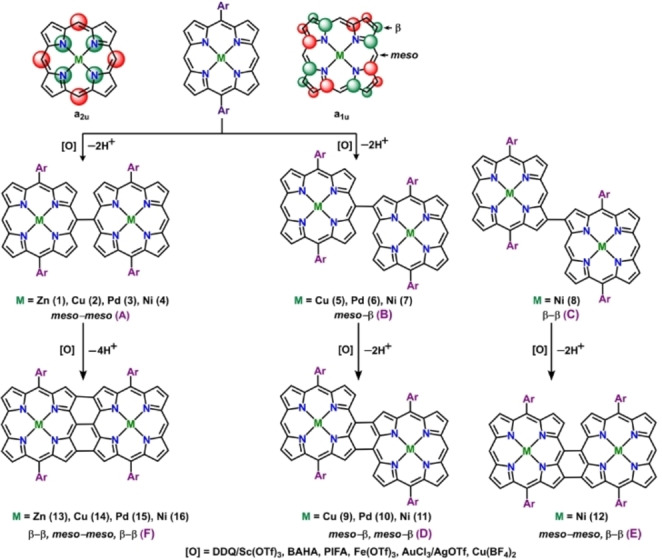
Effect of metals on regioselectivity of the initial C−C bond formation.

In the aforementioned context, directly‐linked bis‐porphyrins have been investigated extensively especially due to their intriguing optical properties.^[16]^ Directly‐linked porphyrins are constructed via covalent bonding between two or more porphyrinic units resulting in a multiple cavity inside a molecular architecture capable of accommodating metal ions. The mode of attachment between porphyrin units typically determines the extent of electronic coupling between the porphyrin chromophores which in effect controls the optical and electronic properties. Usually, covalent linkages among individual porphyrin subunits can be established by installing various spacers of different sizes, shapes, and electronic structures.^[16]^ Although, use of phenylene linkers allows investigation of intramolecular energy and electron transfer satisfactorily, orthogonal arrangements of the linkers result in weak inter‐porphyrin interactions. On the contrary, multiporphyrin arrays bridged by unsaturated groups like alkene or alkyne are rather suitable alternatives for adopting coplanar arrangements between porphyrin sub‐units. Along these lines, linking porphyrin units via multiple covalent bonds either directly or through aromatic spacers is also a viable approach to effectively extend the π‐conjugation. A closer look at the porphyrin structure reveals that direct linkages can be achieved either through free meso or β positions (Scheme 1) or through a combined participation of both. Thus, directly linked porphyrins can be classified mainly into the following different categories; (i) meso‐meso singly linked, (ii) β‐β singly linked, (iii) meso‐β doubly fused, and (iv) β‐β, meso‐meso, β′‐β′ triply fused.

The first example of a meso‐meso linked porphyrin was reported by Osuka and co‐workers in 1997.^[17]^ Following this, meso‐meso linked porphyrin complexes have been extensively studied to investigate their role in molecular wires.^[18]^ However, orthogonal conformation of the neighboring porphyrin units limits their application only to photonic wires capable of transferring excitation energy instead of conducting electrons across the conjugated chain.[^18a^, ^19^] Later, in 2000, the first example of fused porphyrin emerged in the literature involving meso‐β, meso‐β double linkage through C_meso_‐C_β_ bonds.^[20]^ Following this, a β‐β, meso‐meso, β′‐β′ triply fused compound was discovered, which was named as ′porphyrin tape′ attributed to the perfectly planar conformation and conjugation across the network.^[21]^ Over the last two decades, various porphyrin tapes from dimer to dodecamer have been synthesized following different oxidative fusion techniques.^[22]^ These synthetic findings, combined with analyses of various physicochemical properties of the conjugated porphyrins, led to remarkable advances in understanding intriguing features of these frameworks. Moreover, remarkable optical, electrochemical, and electronic features of the conjugated porphyrin arrays prompted further study for various applications for example as a NIR dyes, building block of non‐linear optical materials, photovoltaic devices, multiple charge storage devices, amphiphilic columnar liquid crystals,^[23]^ and so on.

Corrole, a ring‐contracted variant of porphyrin with lower symmetry contains one direct pyrrole‐pyrrole linkage, and three NH hydrogens, allowing stabilization of higher oxidation states of many transition metals, giving rise to intriguing electrochemical properties, and high fluorescence quantum yield as compared to porphyrin counterparts. Ever since the development of facile synthetic protocols for meso triaryl substituted corroles by Gross, Paolesse, Gryko and Tanaka, a rich coordination chemistry of metallocorroles has led to extensive applications in catalysis, sensors, optical materials and medicines. Usually, the extent of electronic communication between porphyrin subunits results in significant alteration in physicochemical properties. Along this line, metal complexes of covalently linked bis‐corroles are of particular interest since oligomerization either through direct linkage or spacers is a useful technique to measure the electronic communication between two individual units. However, the chemistry of directly linked metallocorroles is relatively unexplored as compared to porphyrinic counterpart plausibly due to the lack of effective synthetic strategies.

### 1.1 Scope of the review

In this review article, the recent progress of directly linked bis‐porphyrins/corroles is summarized, with a focus on the electrochemical, spectroscopic, magnetic and electrocatalytic properties. Since several comprehensive reviews regarding the synthesis and properties of the fused porphyrins have already been reported in the literature, the current review will thus focus primarily on the molecular properties of the metal complexes of single linked *meso*‐*meso*, β‐β, *meso*‐ β, β‐β dimers and *meso*‐ β, *meso*‐β‐doubly fused dimers, and β‐β, *meso*‐*meso*, β′‐β′ triply fused dimers. Porphyrin units linked by bridges or spacers, cyclic porphyrin arrays, and porphyrin tapes have been omitted from the discussion as these topics have already been reviewed elsewhere.^[24]^

Osuka and co‐workers recently reviewed directly linked bis‐corroles focussed on the various synthetic routes and structural properties.^[25]^ However, to the best of our knowledge, till date electrochemical, magnetic properties of different types of directly linked bis‐corroles have not been reviewed. In this context, the present article intends to provide a brief overview on different synthetic approaches for the development of corrole dimers along with their electrochemical and magnetic properties.

In the first section of the article, we will focus on a brief overview of synthesis, structural, spectroscopic, and electrochemical properties of the covalently linked metalloporphyrin units followed by their applications in electrocatalytic small‐molecule activation reactions. In the following section, the spectroscopic and electrochemical properties of directly linked metallocorroles will be discussed in detail. This review article is not meant to provide a comprehensive overview of the field. We intend to summarize a brief account of intriguing physicochemical properties of directly linked metalloporphyrinoid oligomers and their applications in several currently relevant research fields.

## 2 Directly Linked Porphyrins

### 2.1 Synthesis

Unique optoelectronic properties combined with extensive applications across interdisciplinary fields fostered the development of improved synthetic protocols to increase yield, solubility, and effective conjugation lengths of oligoporphyrin arrays. Seminal work by Osuka and co‐workers involving oxidative coupling reactions of Zn(II) containing porphyrin monomers in presence of Ag‐salts discussed the facile synthesis of directly linked *meso*‐*meso* porphyrin dimer.^[17]^ Following this report another publication from the same group reported facile synthetic routes for the synthesis of triply‐linked fused diporphyrin following an oxidative double ring closure reaction.^[21]^ Usually, synthesis of covalently linked porphyrin oligomers are carried out using a synthetic sequence of; (a) oxidative *meso*‐*meso* coupling reaction with Ag‐salts followed by chromatographic purification, (b) capping of terminal *meso*‐positions, (c) oxidative fusion reaction with a combination of organic oxidants (DDQ: 2,3‐dichloro‐5,6‐dicyano‐1,4‐benzoquinone/Sc(OTf)_3_) either from the *meso*‐*meso* derivative or directly from the Zn(II)‐monomer to obtain fused porphyrins (or porphyrin tapes). Thus, a primary synthetic strategy for the synthesis of fused porphyrin involves either solution based dehydrogenative coupling of metalloporphyrins with different transition metal salts namely AgX,^[26]^ CuX_2_,^[27]^ WCl_6_,^[28]^ FeX_3_ ^[29]^ and organic oxidants (DDQ, BAHA: tris(4‐bromophenyl)aminiumhexachloroantimonate, PIFA: bis(trifluoroacetoxy)iodobenzene) or electrodeposition.[^20b^, ^30^] In spite of significant progress in the synthesis of expanded porphyrin, poor solubility, requirement of bulky pendant group, or solubilizing substituents have somewhat hindered the widespread applicability of the solution‐based methods. To address this issue, Boscher and co‐workers recently developed a gas phase oxidative Chemical Vapour Deposition (oCVD) technique for the simultaneous synthesis and deposition of fused porphyrin thin films.^[31]^ Since different synthetic strategies related to the preparation of expanded porphyrins have already been reviewed earlier by Osuka and coworkers,^[16]^ therefore a brief overview of the synthetic techniques will be discussed here along with a comprehensive description of the oCVD process.

#### 2.1.1 Solution‐based methods

##### 2.1.1.1 Oxidative C−C coupling

Oxidative C−C coupling reactions facilitated by dehydrogenation of metalloporphyrins in presence of organic oxidants result in the formation of different singly‐, doubly‐ or triply linked bis‐porphyrins. Distribution of the oxidation products usually depends on the various factors like the type of metal ions, substituents at the periphery as well as in the axial positions and reaction medium (solid or gaseous). The most widely accepted mechanistic pathway of oxidative C−C coupling reactions involves nucleophilic attack of the neutral porphyrin at the porphyrin radical cationic species (SOMO). Since, the *meso*‐ position in most of the cases is considered as most electron rich nucleophilic sites, therefore orbital coefficients of the singly‐occupied molecular orbital of porphyrin radical cation govern the regioselectivity of the coupling reaction (Scheme 1, Table 1). Spin population analysis reveals that the symmetry of SOMO of the porphyrin radical cation is either a_1u_ with a larger orbital coefficient at β‐position or a_2u_ with a large orbital coefficient at *meso* position (Scheme 1, Table 1). In the case of electro‐inactive metals like Zn, an unpaired electron in the porphyrin radical cations reside in the π‐orbital of a_2u_ symmetry justifying the formation of *meso*‐*meso* singly linked or triply fused porphyrin dimers (Scheme 1, Table 1). While, in case of Pd, π‐orbital of a_1u_ symmetry hosts the unpaired electron, facilitating formation of *meso*‐β singly linked or doubly fused regioisomers predominantly (Scheme 1, Table 1).^[32]^ In case of Cu, Ni on the other hand, due to the pseudo‐ Jahn‐Teller distortion, orbital symmetry of a_1u_ and a_2u_ orbitals is lowered resulting in a mixture of regioselective products (*meso*‐*meso* or *meso*‐β, Scheme 1, Table 1).

**Table 1:** Oxidation products of metalloporphyrin based on metals, oxidants and solvents.

Entry	Metal	Reagent		Products						Ref.
		Oxidant	Solvent	A	B	C	D	E	F	
1	Zn	AgPF_6_	CHCl_3_	√						[17]
2		PIFA (0.5 equiv.)	CH_2_Cl_2_/CHCl_3_	√	–	–	–	–	–	[33]
3		PIFA (2.5 equiv.)	CH_2_Cl_2_/CHCl_3_	–	–	–	–	–	√	[34]
4		DDQ/Sc(OTf)_3_ (0‐2 equiv.)	toluene	√						[35]
5		DDQ/Sc(OTf)_3_ (5 equiv.)	toluene	–	–	–	–	–	√	[35a]
6		BAHA	CHCl_3_	√	√	–	√	–	√	[20a]
7	Cu	Cu(BF_4_)_2_	CH_3_NO_2_	–	–	–	√	–	–	[36]
8		Cu(ClO_4_)_2_.6H_2_O/K_4_FeCN_6_	CH_3_CN	–	–	–	√	–	√	[27]
9	Pd	DDQ/Sc(OTf)_3_ (2.5 equiv.)	toluene	√	√	√	√	–	√	[37]
10		Fe(OTf)_3_ (3–5 equiv.)	CH_2_Cl_2_/CH_3_NO_2_	–	–	–	√	–	–	[29]
11		BAHA	CHCl_3_	–	–	–	√	–	–	[20a]
12	Ni	AuCl_3_(1‐2 equiv.)/AgOTf(3–10 equiv.)	1,2‐dichloroethane	–	–	–	√	√	√	[37a]
13		Fe(OTf)_3_ (3–5 equiv.)	CH_2_Cl_2_/CH_3_NO_2_	–	–	–	√	–		[29]
14		DDQ (2 equiv.)/Sc(OTf)_3_ (2 equiv.)	toluene	√	–	–	√	–	–	[35a]

DDQ: 2,3‐dichloro‐5,6‐dicyano‐1,4‐benzoquinone, BAHA: tris(4‐bromophenyl)aminium hexachloroantimonate, PIFA: bis(trifluoroacetoxy)iodobenzene, Sc(OTf)_3_: scandium trifluoromethanesulfonate. A: *meso*‐*meso*, B: *meso*‐β, C: β‐β, D: *meso*‐β, *meso*‐β, E: *meso*‐*meso*, β‐ β, F: β‐ β, *meso*‐*meso*, β‐ β.

##### 2.1.1.2 Suzuki coupling

**Scheme 2:**
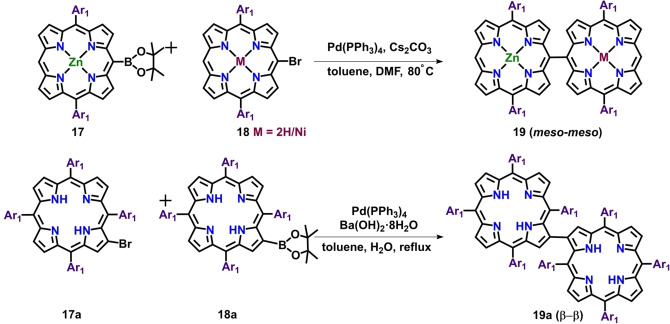
Pd‐catalyzed synthetic protocols for the synthesis of porphyrin dimer.

Taking into account the advantage of oxidative coupling reaction described above, Zheng and co‐workers described a synthetic protocol for the synthesis of *meso*‐*meso* linked Zn(II)‐Ni(II) hybrid porphyrin dimer via Suzuki coupling reaction of Zn(II) and Ni(II) monoporphyrin units (Scheme 2).[^34^, ^38^] Furthermore, this singly‐linked dimer was shown to undergo PIFA‐mediated oxidative fusion reaction to form respective triply fused hybrid bis‐porphyrins. Similarly, Senge, Bringmann and co‐workers synthesized β‐β directly‐linked porphyrin dimers in appreciable yield following Suzuki‐Miyaura cross‐coupling reaction.^[39]^

#### 2.1.2 Oxidative chemical vapor deposition method (oCVD)

**Scheme 3:**
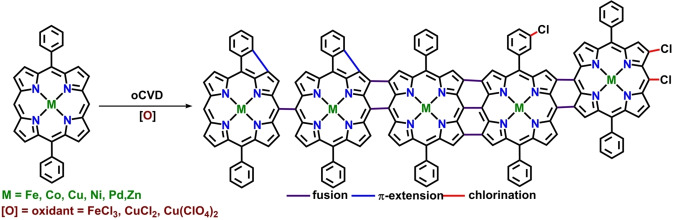
Illustration of different reactions occurring during the oCVD reaction. Reproduced with permission from Ref. [31d] ©2020, the Royal Society of Chemistry.

The oxidative chemical vapor deposition method relies on vapor phase delivery of porphyrin monomer and oxidant to a surface for subsequent synthesis and deposition of conjugated polymer thin films on a variety of substrates such as silicon, glass, and printer paper. Boscher, Heinze and co‐workers recently reported the synthesis, deposition, and π‐doping of porphyrin thin films using the oCVD approach (Scheme 3).[^31a^, ^31b^, ^31c^, ^31d^] One of the advantages of the oCVD approach over solution‐based methods is that specific functionalization of the porphyrin monomer is not required, thereby commercially available or easy‐to‐synthesize inexpensive porphyrins can be used for the fusion of multiple porphyrin rings. Availability of free *meso* and β‐ position, and excellent hydrolytic stability even in presence of acid lead to the selection of nickel(II) 5,15‐diphenyl porphyrin (NiDPP) as a preferred monomer for the oCVD process along with the FeCl_3_ as an oxidant. Custom‐built stainless‐steel chamber equipped with two crucibles for simultaneous sublimation of porphyrin monomer and oxidant were used to carry out the oCVD reaction under reduced pressure (10^−3^ mbar). Substrates were placed atop a heated stage placed 20 cm above the crucibles to obtain 200 nm thick and strongly colored dark green coatings of fused porphyrin oligomer in contrary to the orange‐colored reference NiDPP coating formed in absence of oxidant. The versatility and potential of the oCVD method were demonstrated from the use of a wide range of substrates like printer paper sheets, microscope glass slides, silicon wafers, and commercial organic field‐effect transistor chips. Laser desorption ionization high‐resolution mass spectrometry (LDI‐HRMS), and gel permeation chromatography (GPC) along with absorption spectroscopic techniques were used to confirm molecular identity of the porphyrin oligomers, while the morphology of the thin films was assessed by scanning electron microscopy (SEM) and atomic force microscopy (AFM). The practicality of the method was further proven by successive reports from the same group with different transition metals (Fe, Co, Cu, Pd, Ni), and the tolerance of functional groups in the presence of the volatile oxidant FeCl_3_ for dehydrogenative coupling.[^31a^, ^31b^, ^31c^, ^31d^] One of the unique advantages of the oCVD method is that even side reaction products (Scheme 3) such as chlorinated porphyrin and π‐conjugated porphyrin oligomers (formed from the intramolecular ring fusion) could also be used as a means to provide extra stability to the film in addition to increasing the π‐conjugation.

#### 2.1.3 Electrochemical techniques

**Scheme 4:**
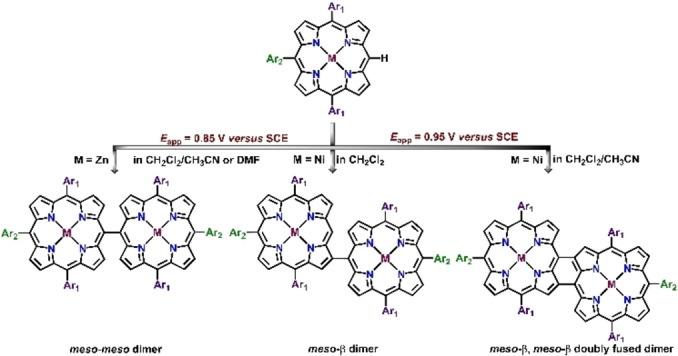
Product distribution in electrochemical oxidative C−C coupling of porphyrin monomer.

**Scheme 5:**
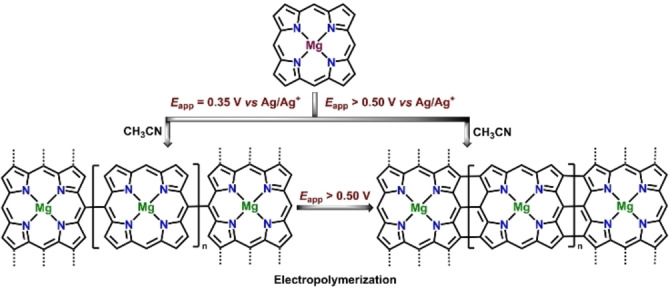
Electrochemical oxidation of Mg‐porphine into *meso*‐*meso* singly‐linked and triply‐fused oligomers.

Electrochemical oxidation is a sustainable alternative to oxidative chemical transformation for the synthesis of directly linked *meso*‐*meso* or triply‐fused porphyrin dimers. Osuka and co‐workers for the first time investigated electrochemical oxidation of a different *meso*‐free metalloporphyrin monomers for the synthesis of directly linked porphyrin dimers.^[40]^ In 2012, Dimé and co‐workers revisited the electrochemical synthesis of Zn‐containing meso‐*meso* dimer from respective zinc‐5,15‐p‐ditolyl‐10‐phenylporphyrin monomer at its first oxidation potential in DMF with 2,6‐lutidine as a base.^[30]^ They proposed a two‐step oxidation of the Zn‐monomer as a necessary prerequisite for the formation of dimer with first period of oxidation generating corresponding cation radical of the dimer followed by a second oxidation to yield the neutral species. In a follow up work, they further exemplified the role of solvents, the electrode cell configurations and the number of electrons abstracted towards formation of different coupling products in the case of 10‐phenyl‐5,15‐di‐p‐tolylporphyrin Ni(II) complex.^[41]^ While electrolysis in CH_2_Cl_2_/CH_3_CN solution in a three electrode configuration cell yielded *meso*‐β/*meso*‐β doubly fused dimer, similar reaction conditions with CH_2_Cl_2_ resulted in the formation of *meso*‐β singly linked dimer (Scheme 4).^[41]^ Later in 2010, Devillers and co‐workers conducted an electrochemical polymerization reaction utilizing Mg(II) complexes of unsubstituted porphyrin, where they showed that Mg‐porphine upon electrolysis at its first oxidation potential led to the formation of *meso*‐*meso* dimer along with higher coupling products.^[42]^ This porphyrin based polymer deposited on the electrode surface results in the formation of singly linked (*meso*‐*meso*) polymer at an applied potential <0.40 V (versus Ag/AgCl reference electrode), while application of potential >0.50 V leads to the formation of corresponding triply fused porphyrin polymers (Scheme 5).

### 2.2 Electrochemistry

**Figure 1:**
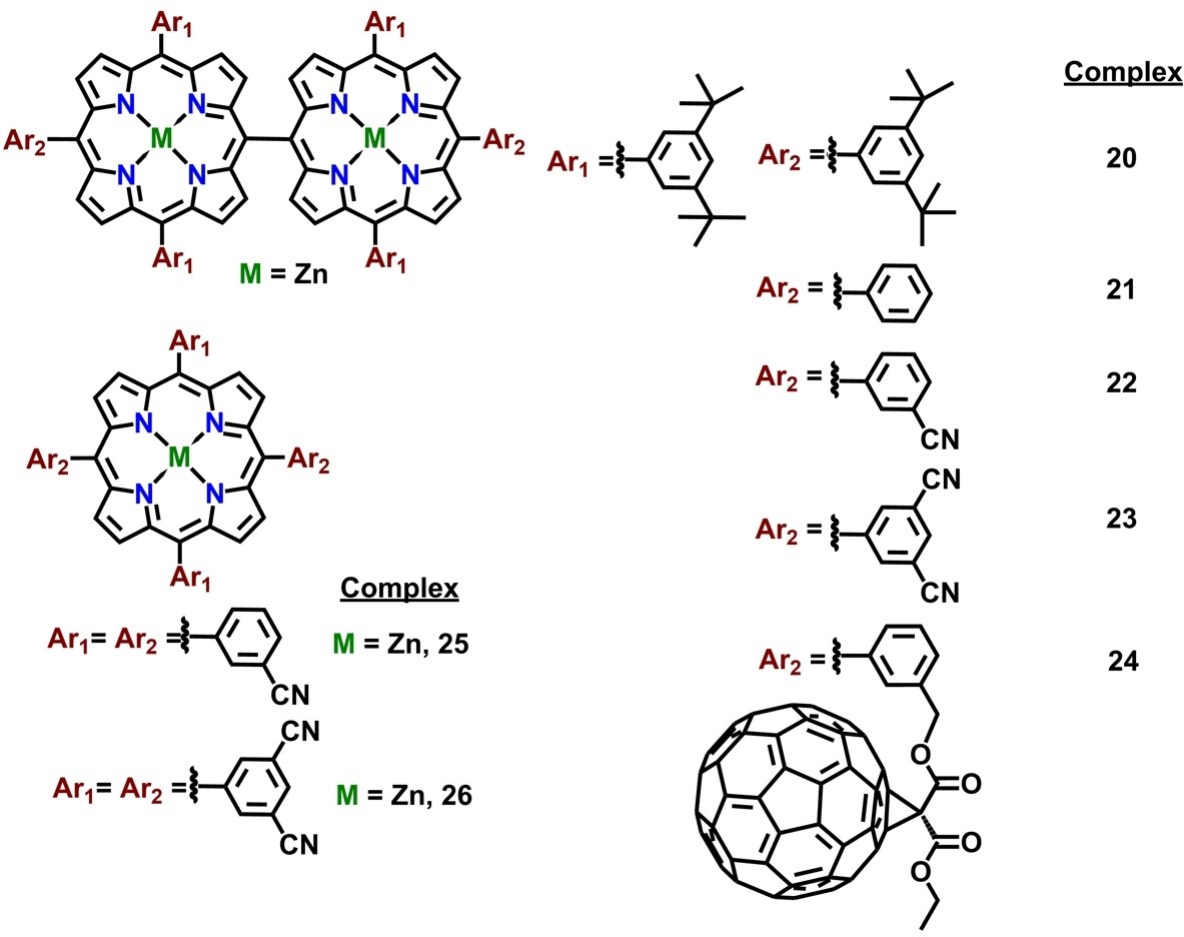
*meso*‐*meso* linked metalloporphyrins investigated for electrochemical properties.

**Figure 2:**
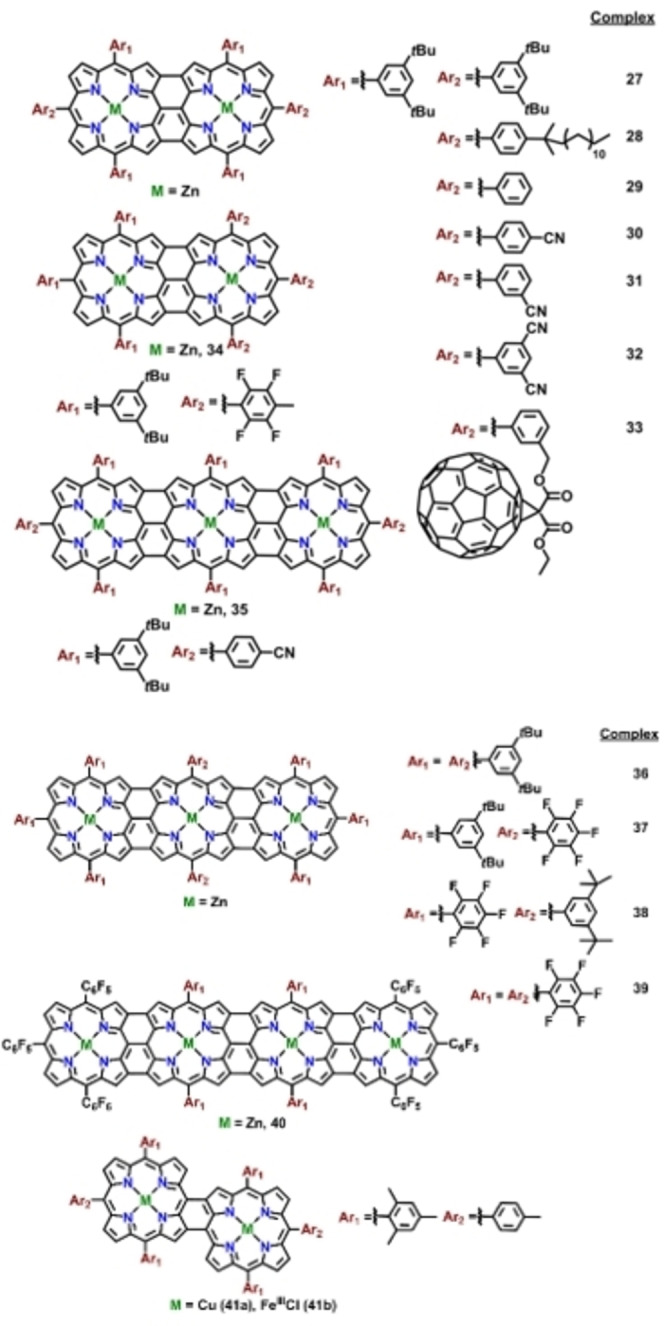
Fused metalloporphyrins investigated for electrochemical properties.

Electrochemical investigation of the directly linked bis‐porphyrins (*meso*‐*meso* or triply fused, Figures 1 and 2) reveals critical information regarding the nature of interaction between the metal‐ions in the subunits. Practically, all the reported metal complexes of covalently linked multiporphyrins are electroactive and undergo successive redox processes involving electron abstraction or addition centered at the metal ions or the conjugated π‐ring of the macrocycle. Naturally, the number of the redox processes, half‐wave potentials, and the site of electron transfer (metal, macrocyclic π‐ring or the axial ligand) rely on various factors; (i) type of the macrocycle, (ii) potential range of solvents used, (iii) oxidation state of the central metal ions, (iv) type of functional groups at β‐ or *meso*‐ positions of the macrocyclic ring, (v) type of metal ions and axial ligands.

In general, metal complexes of directly linked bis‐porphyrins similar to metalloporphyrin monomers undergo stepwise 1e^−^/2e^−^ oxidation or reduction generating π‐cation radicals/dications or π‐anion radicals/dianions. However, due to extended conjugation across the multiple porphyrin units, directly linked porphyrin oligomers exhibit complicated redox situations depending on various external factors. Electrochemical tools like cyclic voltammetry and differential pulse voltammetry are used to obtain necessary information regarding the electrochemical processes as for example half‐wave potentials (*E*_1/2_) of the redox processes, and the difference in peak potentials (Δ*E*) to calculate electrochemical HOMO‐LUMO gaps. This set of data are then utilized as a diagnostic tool for a comparison between different metalloporphyrins.

In case of metalloporphyrins containing non‐electroactive metal centers like Zn^II^, redox processes are mostly confined within the π‐conjugated ring of the macrocycle. Likewise, Ni^II^ or Cu^II^ containing bis‐porphyrins also exhibit identical electrochemical behaviour suggesting predominant contribution of π‐systems of the macrocycle as opposed to metal center towards electron transfer processes. The reversible or irreversible nature of the redox waves involving porphyrin π‐systems thus depends on the nature of the macrocycle, substituents, and the degree of stability of the respective cationic or anionic species in solution. The origin of the irreversible redox processes can be attributed to the tendency of the electrogenerated species (oxidized/reduced) to undergo further chemical reactions for example intermolecular coupling between oxidized and reduced units of two porphyrin moieties, the reaction between solvent and product of electron transfer, etc.

**Figure 3:**
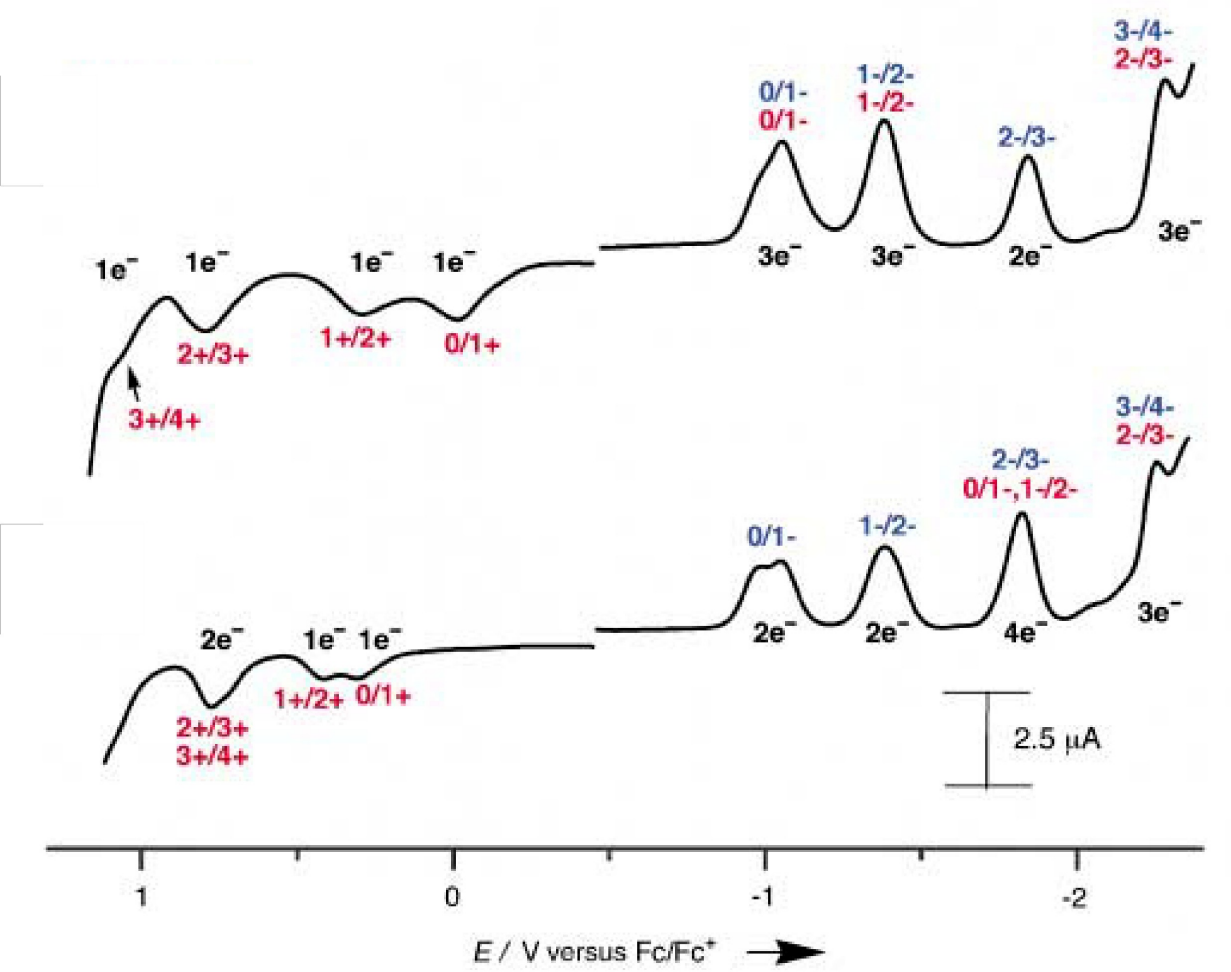
Differential pulse voltammograms of C_60_ conjugated fused porphyrin (top) and *meso*‐*meso* porphyrin (bottom). Reproduced with permission from Ref. [43a] ©2003 Wiley‐VCH GmbH.

Singly‐linked *meso*‐*meso* porphyrin dimers exhibit characteristic electrochemical behaviour displayed by two monomeric units connected in an orthogonal fashion. Subsequently, number of redox processes observed in the porphyrin dimers are sometimes doubled as compared to the respective monomeric analogues attributed to the number of connected electroactive chromophores. Analysis of electrochemical potentials in a series of *meso*‐*meso* porphyrin dimers reveal that replacement of electron‐donating *tert*‐butyl group by π‐accepting ‐CN led to the significant shift in reduction and oxidation potential respectively from complexes **20**→**23** (Figure 1 and Table 2). In comparison to the metalloporphyrin monomers **25** and **26**, number of redox processes in the *meso*‐*meso* forms are almost doubled (Table 2).[^42^, ^43^] On the other hand, conjugation of the two C_60_ moieties to the singly linked porphyrin dimers resulted in a multiple reversible redox process with an involvement of as many as 15 electrons (Table 2, Figure 3). Thus, *meso*‐*meso* bis*‐*porphyrins exhibit electrochemical behaviour more or less identical to the monomeric, which is also reflected from the almost identical energy differences between HOMO and LUMO orbitals (Table 2), with the only difference being the number of redox processes.

**Table 2:** Electrochemical potentials and HOMO‐LUMO gaps (Δ*E*) for the triply linked porphyrin arrays.

Complexes	Oxidation					Reduction						Δ*E* ^[a]^ [eV]	Ref.
	$E_{ox5}^{1/2}$	$E_{ox4}^{1/2}$	$E_{ox3}^{1/2}$	$E_{ox2}^{1/2}$	$E_{ox1}^{1/2}$	$E_{red1}^{1/2}$	$E_{red2}^{1/2}$	$E_{red3}^{1/2}$	$E_{red4}^{1/2}$	$E_{red5}^{1/2}$	$E_{red6}^{1/2}$		
20^[b]^	–	0.57	0.36	‐0.06	−0.27	−2.32	−2.42	−2.74	‐	‐	‐	2.05	[43a]
22^[b]^	0.66	0.34	0.28	0.03	−0.06	−2.17	−2.29	−2.64	‐2.83	‐	‐	2.11	[43a]
23^[b]^	0.70	0.53	0.34	0.07	−0.03	−2.14	−2.26	−2.55	‐2.77	‐	‐	2.11	[43a]
24^[b]^	–	0.38	0.27	0.04	−0.09	−1.44	−1.87	−2.30	‐	‐	‐	1.35	[43c]
25^[b]^	–	–	–	0.23	−0.05	−2.12	−2.49	−2.70	‐	‐	‐	2.07	[43a]
26^[b]^	–	–	–	0.29	0.01	−2.11	−2.44	−2.80	‐	‐	‐	2.12	[43a]
27^[c]^	–	1.03	0.77	0.28	−0.03	−1.13	–	–	–	–	–	1.10	[20a]
28^[c]^	–	–	–	–	0.21	–	–	–	–	–	–	–	[21]
29^[b]^	–	–	–	−0.20	−0.39	−1.59	−1.83	–	–	–	–	1.20	[34]
30^[d]^	–	–	0.75	0.37	0.12	−1.12	−1.58	−2.42	–	–	–	1.24	[45]
31^[b]^	–	–	0.43	−0.08	−0.45	−1.53	−1.72	−2.63	–	–	–	1.08	[43a]
32^[b]^	–	0.62	0.40	−0.13	−0.33	−1.43	−1.67	−2.60	–	–	–	1.10	[43a]
33^[b]^	–	0.63	0.36	−0.12	−0.43	−1.45	−1.86	−2.33	−2.75	–	–	1.02	[43c]
34^[b]^	–	0.74	0.48	0.01	−0.24	−1.43	−1.67	‐	–	–	–	1.19	[20a]
35^[b]^	–	–	0.09	−0.30	−0.45	−1.32	−1.47	−2.23	–	–	–	0.87	[45]
35^[d]^	–	–	–	0.28	0.02	−0.88	−1.12	−1.90	‐2.17	–	–	0.90	
36^*b*^	–	0.24	–0.01	−0.41	−0.59	−1.57	–	–	–	–	–	0.98	[43b]
37^*b*^	–	0.31	0.07	−0.29	−0.46	−1.27	−1.46	−2.12	‐2.42	–	–	0.81	[43b]
38^*b*^	–	–	–	–	−0.39	−1.21	−1.43	–	–	–	–	0.82	[43b]
39^*b*^	–	–	–	–	−0.29	−1.15	−1.35	–	–	–	–	0.86	[43b]
40^*b*^	–	–	–	−0.23	−0.41	−1.06	−1.18	−1.36	–	–	–	0.65	[43b]
41^*c*^	–	–	–	0.56	0.34	−1.23	−1.52	‐	–	–	‐	1.57	^[45]^
42^*e*^	–	–	–	–	–	−0.60	−0.72	−1.21	−1.46	−2.11	−2.40	–	^[44]^

^*a*^Δ*E* (eV): *E*_ox1_−*E*_red1_=Electrochemical HOMO‐LUMO gap. ^*b*^measured in CHCl_3_ with Ag/AgClO_4_ reference electrode (reported vs. Fc/Fc^+^ considering standard *E*_1/2_ of redox couple in CHCl_3_) .^*c*^in CH_2_Cl_2_ vs. Fc/Fc^+^ with 0.1 M *n*Bu_4_NPF_6_. ^*d*^in THF vs. Fc/Fc^+^ with 0.1 M *n*Bu_4_NPF_6_. ^*e*^ in DMF vs. Fc/Fc^+^ with 0.1 M *n*Bu_4_NPF_6_.

Triply fused derivatives display clearly defined multielectronic redox processes in contrast to the poorly defined, overlapping redox waves in singly linked *meso*‐*meso* derivatives. Comparison of the first oxidation potential between monomer (**25** and **26**, Figure 1), *meso*‐*meso* (**20**–**23**, Figure 1) and triply fused derivatives (**27**–**34**, Figure 2) clearly indicate significant lowering of the potential in case of fused dimer attributed to the extensive conjugation across the porphyrin frameworks as opposed to the *meso*‐*meso* diporphyrin analogues having an orthogonal conformation between individual porphyrin units. The decrease in first oxidation potential becomes more pronounced upon increasing the number of porphyrin units as observed in case of complexes containing trimeric (**35**, Figure 2 and Table 2) and tetrameric porphyrin tapes (**40**, Figure 2 and Table 2).[^43b^, ^43c^] In line with the argument regarding perturbation in redox potentials with increase in conjugated units in porphyrin tapes, tetrameric porphyrin tape furnished five redox processes within a narrow potential window of 1.13 V.[^43b^, ^43c^] As expected, these trimeric and tetrameric porphyrin tapes in presence of electron rich (*tert*‐butyl) or electron‐deficient (pentafluro) functional groups display negative or positive shift in the electrochemical potential.[^43b^, ^43c^] The triply fused porphyrin dimer containing covalently attached C_60_ spheres in the peripheral positions display multi‐electron redox processes within a narrow potential window with a significant cathodic shift of the first oxidation potential as compared to respective singly linked *meso*‐*meso* derivative (Table 2, Figure 3).

Moore and co‐workers in a recent publication reported electrochemical analysis of a binuclear Fe(III) doubly fused porphyrin and compared its properties with corresponding Fe(III)‐monomer.^[44]^ They have shown that bis‐metalloporphyrin can store upto six electrons as evident from six reversible one electron reduction processes in DMF. In the contrary, corresponding metalloporphyrin monomer display three reversible one‐electron reduction processes under identical experimental conditions. This difference in electron transfer can be attributed to the π‐extension of the ligand scaffold allowing delocalization across the multimetallic assembly.

Electrochemical HOMO‐LUMO energy gap calculated from the redox potentials further highlights the differences between monomer and the two different types of dimer. While electrochemical HOMO‐LUMO gap in *meso*‐*meso* porphyrin dimer (**23**, 2.11 eV, Table 2) is almost similar to the respective monomer (**26**, 2.12 eV, Table 2), the same for the respective triply fused dimer (**32**, 1.10 eV, Table 2) is significantly low, which is primarily responsible for large cathodic/anodic shift of the first oxidation/reduction potentials. This difference in behaviour between two dimeric complexes is a further testament to extensive delocalization in case of planar triply fused dimeric complexes. Varying electronic properties of the functional groups in the peripheral position of the fused dimer affect the redox potential in a similar manner as observed in case of the *meso*‐*meso* linked complexes.

Hence, a general conclusion drawn from this comparative survey of electrochemical behaviour is that fused porphyrin dimers due to the planar structure facilitate extensive delocalization of electrons within the porphyrin ligand framework, thus reducing the electrochemical HOMO‐LUMO gap resulting in multi‐electronic redox processes within a narrow potential window. On the contrary, singly linked *meso*‐*meso* porphyrin dimers display electrochemical properties which are more or less similar to monoporphyrin derivatives. However, the number of redox processes in case of *meso*‐*meso* complexes are far greater than respective monomeric analogues, mirroring doubling the number of orthogonally connected individual porphyrin units.

### 2.3 Electrocatalytic and Photocatalytic Proton reduction

Fused metalloporphyrins, owing to extensive delocalization across extended multimetallic scaffolds are capable of storing multiple redox equivalents within the frameworks. These types of frameworks therefore can act as an electron reservoir, which could possibly be exploited in electrocatalytic small molecule activation reactions. Moore and co‐workers in this context reported a bimetallic Cu‐fused porphyrin complex (Cu_2_‐fused) which exhibited superior electrocatalytic performances in proton reduction reaction over the respective monomeric analogue (Cu‐mono) under an identical experimental setup.^[46]^ The bimetallic complex was shown to undergo oxidation or reduction at less applied bias potential as compared to the nonfused porphyrin complex.

**Figure 4:**
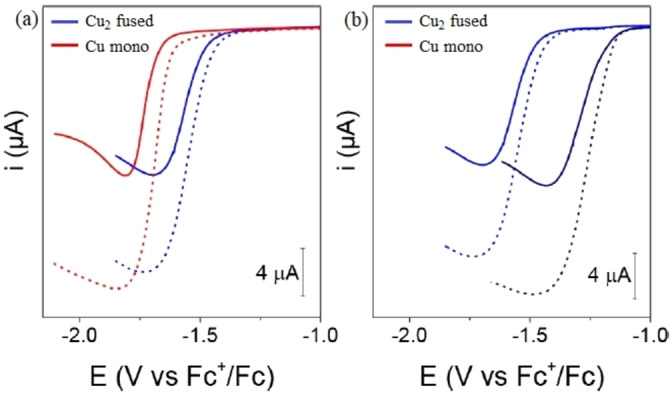
Linear sweep voltammograms of (a) 0.01 mM and (b) 0.1 mM of fused and monomeric metalloporphyrin in the presence of 16.25 mM (solid) and 32.5 mM of acid concentrations (Condition: CH_2_Cl_2_, 0.1 M *n*Bu_4_PF_6_, glassy carbon electrode, scan rate: 100 mV/s) Reproduced with permission from Ref. [46] ©2018 American Chemical Society.

Electrochemical properties of the 0.01 mM dichloromethane solution of fused metalloporphyrin were evaluated with *n*Bu_4_PF_6_ as an electrolyte, TFA (TFA=trifluoracetic acid) as a proton source using an electrochemical cell equipped with glassy carbon working electrode in a three‐electrode configuration. Catalytic activity of the fused metalloporphyrin catalyst was compared with the monomeric analogue from various control electrochemical experiments under identical experimental conditions. Titration of 0.01 mM solutions of Cu_2_‐fused porphyrin complex in presence of different concentrations of TFA (16.25 mM ‐ 84.5 mM) furnished irreversible catalytic wave with a onset potential of −1.56 V. Linear sweep voltammetric experiments performed with similar concentrations (0.01 mM) of both the catalysts (fused and nonfused) and acid (16.25 or 32.5 mM) revealed that bimetallic copper fused porphyrin furnished equivalent amount of catalytic current of 5 μA at potentials 170 mV positive than that of respective monomeric analogue, which remains unchanged even after doubling the concentration (0.02 mM) of nonfused metalloporphyrin. This indeed suggests easy accessibility of the catalytically active reduced species responsible for facilitating electrochemical HER in case of doubly fused dimer compared to nonfused monomeric counterpart. Ease of reduction in case of fused bimetallic porphyrin is found to be consistent with significant improvement in overpotential for given turnover frequency. The desired S‐shaped voltammograms observed at relatively higher concentration of acid (>19.5 mM) and scan rate ≥600 mV further implied kinetically controlled hydrogen evolution reaction (HER), opposed to the diffusion of substrate protons (Figure 4).

Kinetic analysis of the HER process yielded a rate constant (*k*_obs_) of 2.2×10^6^ s^−1^ which is among the highest reported values when compared with several literature reported molecular catalysts. Further, rotating ring disk electrode analysis for quantification of evolved H_2_ gas in combination with various control electrochemical experiments like acid stability studies, effect of concentration of catalyst and proton source highlighted robust nature of the binuclear Cu‐containing fused porphyrin catalyst towards electrochemical HER over the respective monomeric analogues.

Recent publications from Boscher and co‐workers reported on a doubly fused Cu(II)‐containing porphyrin thin film which was shown to be an active electrocatalysts and photocatalysts towards heterogeneous HER.^[47]^ The fused porphyrin thin films notably reduced the overpotential for HER in comparison to the respective nonfused porphyrin thin films suggesting efficient separation and storage of multiple charges due to extension of π‐electron conjugation. Further studies involving different substituents at the peripheral position (phenyl and mesityl) of the fused porphyrin suggested influence of functional groups on the superior catalytic activity of the bis‐metalloporphyrin thin films. It was shown that intramolecular dehydrogenative coupling between free *ortho* and b‐ positions of the phenyl substituent and porphyrin macrocycle respectively reduces overpotential for HER, unlike the corresponding mesityl substituted porphyrin thin films due to unavailability of the ortho positions.

Thus, bimetallic fused porphyrin architecture on account of conjugation across the porphyrin π‐system permits easy access to the catalytically active reduced species resulting in favourable catalytic properties towards electrochemical proton reduction reaction over the analogous nonfused monoporphyrin complex. This indeed open up new avenues for modulation of electrochemical potential of porphyrin moieties instead of relying on electron withdrawing/donating functional groups. Enhanced catalytic properties of the fused bimetallic porphyrin moieties thus indicate a promising strategy for designing efficient electrocatalysts for small molecule activation.

### 2.4 Optical properties

One of the prominent features of the porphyrin derivatives are their characteristic electronic absorption spectrum comprising of two distinct set of bands namely ′Soret′ and ′Q‐bands′ in the near‐UV and visible regions respectively. Usually, relative position of these bands are affected by the extent of conjugation, type and position of substituents, identities of metal center and symmetry of the porphyrin. The origin of these two bands can be understood based on the successful ′Four‐orbital′ theory proposed by Gouterman, explaining the role of charge delocalization on the absorption spectra of porphyrins.^[48]^

**Figure 5:**
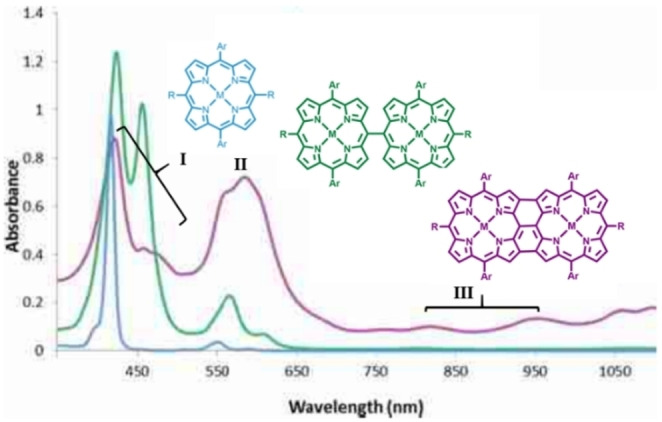
Absorption features of the monomer (blue), *meso*‐*meso* linked porphyrin dimer, and fused dimer in solution. Reproduced with permission from Ref. [35a] with permission from ©2013 Wiley‐VCH GmbH.

In a typical porphyrin monomer, absorption region of the intense Soret band remains in between 350–500 nm depending on the substituents at the β‐ or *meso*‐positions, while Q‐band of weaker intensity appears in the 500–700 nm range. Two different series of porphyrin dimer (singly‐linked or doubly/triply‐linked fused species) display absorption features based on the extent and type of interactions between connected porphyrin subunits. Directly linked (*meso*‐*meso*/*meso*‐β/β‐β) porphyrin dimers usually exhibit splitting of the Soret bands due to the large excitonic coupling among the individual porphyrin units, while spectral changes pertaining to Q‐band display slightly broadened features (Figure 5).[^35a^, ^49^] The origin of the splitting of Soret bands in case of *meso*‐*meso* linked porphyrin can be ascribed to the coulombic interactions between the transition dipole moment indicating disruption of the electron delocalization across the π‐conjugated system which can be qualitatively accounted for by the orthogonal conformation of the neighboring porphyrins. Fused porphyrin dimers on the other hand display significantly altered absorption profile as compared to the directly linked and monomeric counterparts. Although the relative position of the Soret bands (band I and II) remains largely unchanged, excitonic coupling between the porphyrin units results in the splitting of the inherent Soret bands into two bands. Furthermore, the peak position of the Q‐band (band III) undergoes significant bathochromic shift mainly due to extensive π‐conjugation across the covalently linked porphyrin units resulting in reduced energy gap between the highest occupied (HOMO) and the lowest unoccupied (LUMO) molecular orbitals. This reduced energy gap between frontier molecular orbitals causes substantial red shift of the Q‐bands into the near‐infrared regions (Figure 5). With the increase in the number of porphyrin units, band III progressively shifts to the near‐IR/IR region with tails extending beyond 2000 nm as observed in case of a dodecameric porphyrin tape.^[44]^ Thus, optical properties of the conjugated porphyrins serve as important markers to differentiate between differently linked bis‐porphyrins.

### 2.5 Magnetic properties

**Figure 6:**
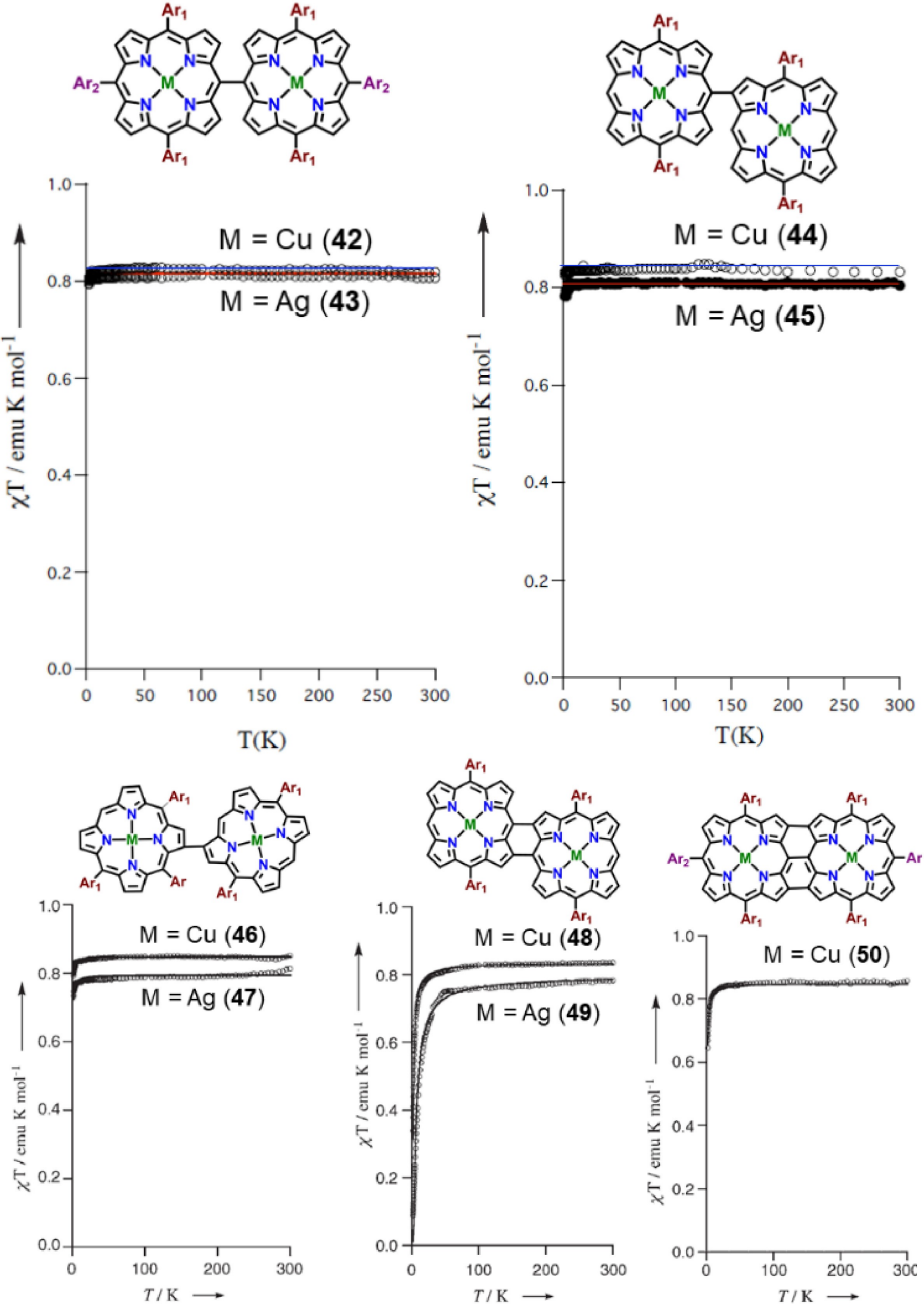
Variable temperature magnetic susceptibility measurements between 2–300 K. Reproduced with permission from Ref. [22a] ©2005 Wiley‐VCH GmbH.

Covalently linked oligoporphyrins, owing to their large electronic interactions between metal centers, have been exploited in long‐range magnetic coupling interactions. Osuka and co‐workers investigated a series of Cu‐ and Ag complexes of singly‐ (*meso*‐*meso*, *meso*‐β, β‐β), and doubly‐ (*meso*‐*meso*, β‐β), triply‐ (β‐β, *meso*‐*meso*, β‐β) linked porphyrin dimers for magnetic communication between distant metal centers.^[22a]^ They reported that in case of *meso*‐*meso*, *meso*‐β singly and doubly‐ (*meso*‐*meso*, β‐β) linked metalloporphyrin dimers, magnetic susceptibility (*χ*T) remained invariably constant in the temperature range 2–300 K with a constant value of 0.8 emu K mol^−1^. EPR spectroscopic analysis combined with temperature ‐dependent magnetic studies led them to conclude two magnetically uncoupled spin doublets in the singly linked porphyrin dimers. In contrast, triply‐ (β‐β, *meso*‐*meso*, β‐β) linked porphyrin dimers for both the metal complexes were shown to exhibit temperature‐dependent magnetic susceptibility (*χ*T) value below 20 K with a final value of 0.34 emu K mol^−1^ at 2 K, indicating weak antiferromagnetic coupling between the two‐individual metal centers mediated by the intervening bridge (Figure 6). Similar behaviour was noticed in the case of β‐β ‐linked porphyrin dimers thus signifying the critical role of direct β‐β bond in long‐range antiferromagnetic coupling (Figure 6). EPR spectroscopic analysis in these cases also confirmed the presence of magnetically interacting metal centers. Furthermore, spin population analysis of the model substances through DFT calculations suggested the presence of unpaired electrons in the d_x_^2^_‐y_^2^ orbitals of Cu‐ and Ag‐metal centers across the β‐positions emphasizing significance of β‐β bond in long‐range antiferromagnetic coupling displayed by fused metalloporphyrin dimers. The favorable long range electronic communication across β‐β ‐bonds in bisporphyrins indeed warrants further analysis with different electroactive metal centers.

### 2.6 Electrical conductivity

**Figure 7:**
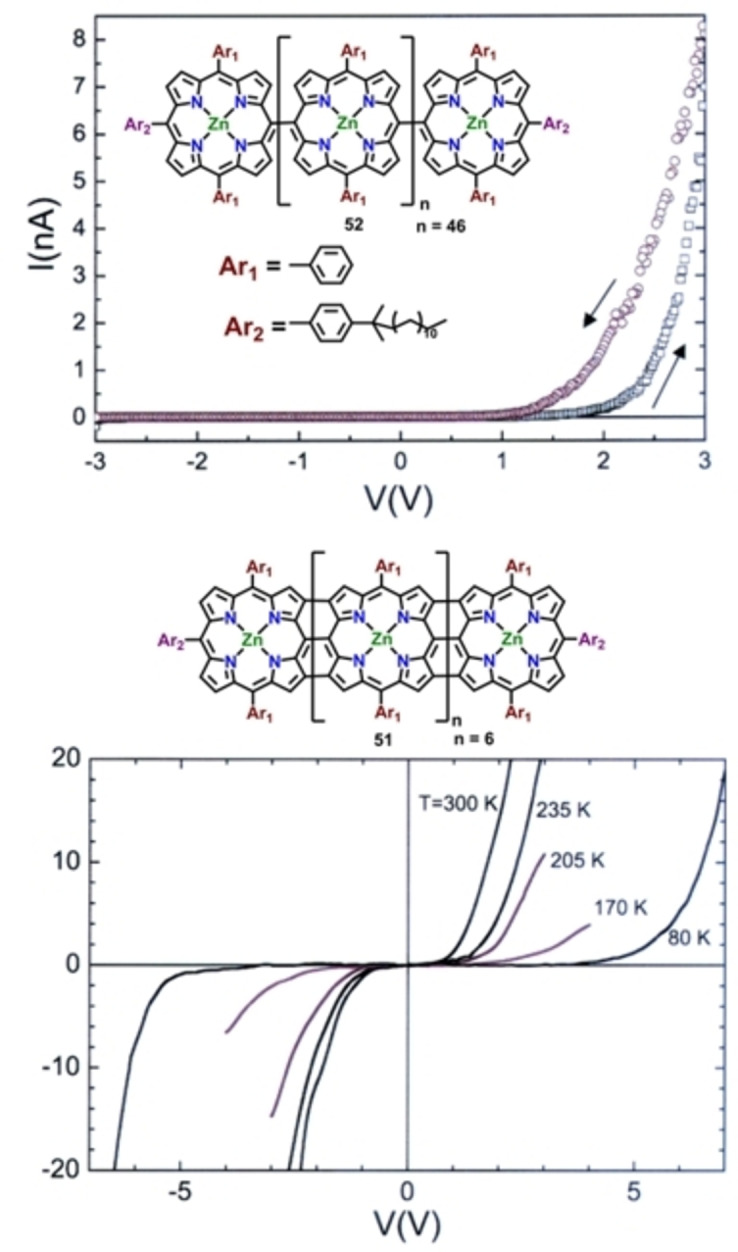
*I‐V* curves of (a) *meso*‐*meso* and (b) fused porphyrin arrays. Reproduced with permission from Ref. [50], ©2018 American Chemical Society.

The overlap of π‐conjugated molecular orbitals of the covalently linked oligoporphyrin arrays have given impetus to the investigation of their electrical transport properties. In this context, Kim and co‐workers demonstrated the influence of the electronic interactions among neighbouring porphyrin molecules on electrical conductivity.^[50]^ For this purpose, they analysed conductive properties of two different sets of oligoporphyrin arrays; a *meso*‐*meso* linked Zn(II) porphyrin array consisting of 48 porphyrin units and triply linked fused Zn(II) porphyrins comprising of 8 porphyrin units. Analysis of electrical transport properties of these two molecules has been carried out via two types of Au/Ti‐nanoelectrodes of different lengths and spacing following the electrostatic trapping method to establish a connection between metal electrodes and porphyrin arrays. *I‐V* curve of the *meso*‐*meso* linked moiety at room temperature displayed diode‐like behavior and the hysteresis dependent on the direction of voltage sweep attributed to the conformational heterogeneity arising out of orthogonal conformation between individual porphyrin subunits (Figure 7). On the contrary, *I‐V* curve of the fused porphyrin moiety exhibited symmetric response without any hysteresis (Figure 7). Furthermore, the fused porphyrin arrays displayed superior conductivity and smaller resistance as compared to the analogous *meso*‐*meso* derivatives implying a smaller bandgap. Later, Boscher, Heinze and co‐workers investigated electrical conductivity of the porphyrin oligomers synthesized via oxidative chemical vapor deposition methods (oCVD) using conductive atomic force microscopic technique (cAFM). Influence of various factors like choice of metal, oxidants, substituents at the periphery on the conductivity has been evaluated. From local electron‐current distribution they concluded that Zn(II) containing triply linked porphyrin oligomer due to their flat, tape‐shaped structure, and extensive π–π stacking attributed to planar conformation, induces excellent conductivity in contrast to the respective directly linked (*meso*‐ *meso*, *meso*‐β, b‐b) multiporphyrin arrays. Another report from the same group demonstrated the effect of intramolecular dehydrogenative cyclization reaction on conductive properties of the porphyrin oligomers.[^31a^, ^31b^, ^31c^, ^31d^] Theoretical investigation combined with various experimental evidences pointed towards extensive π–π stacking in the case of phenyl and tert‐butyl (at 3,5 position of the phenyl ring) substituted Ni(II)‐containing diphenyl porphyrin oligomers imposing planarity of the macrocyclic ring resulting in increased conductivity. On the contrary, mesityl, and dodecyloxyphenyl (at 2,6 position of the phenyl ring) substituted derivatives have been observed to hinder the π–π stacking interactions among the molecular planes of the porphyrin frameworks preventing the adoption of planar conformation resulting in diminished conductivity.

## 3 Directly Linked Corroles

### 3.1 Synthesis

Directly linked corrole dimers can be classified into following different categories: (i) β‐β singly linked (3‐3′, 2‐2′ linkage), (ii) *meso*‐*meso* linked: it is to be noted that lower symmetry of corrole facilitates the formation of *meso*‐*meso* linked corrole dimers with different linkages (5‐5′, 10‐10′ linkage) resulting in different torsional angle around the *meso‐meso* bond; (iii) doubly linked corrole dimers (3‐5, 2‐18 linkage); (iv) triply linked corrole dimers. Among these, doubly linked and triply linked corroles are most studied because of their unique redox interconversions between aromatic 3NH‐ type dimers and non‐aromatic 2NH‐ type dimers.

#### 3.1.1 β‐β singly linked

**Scheme 6:**
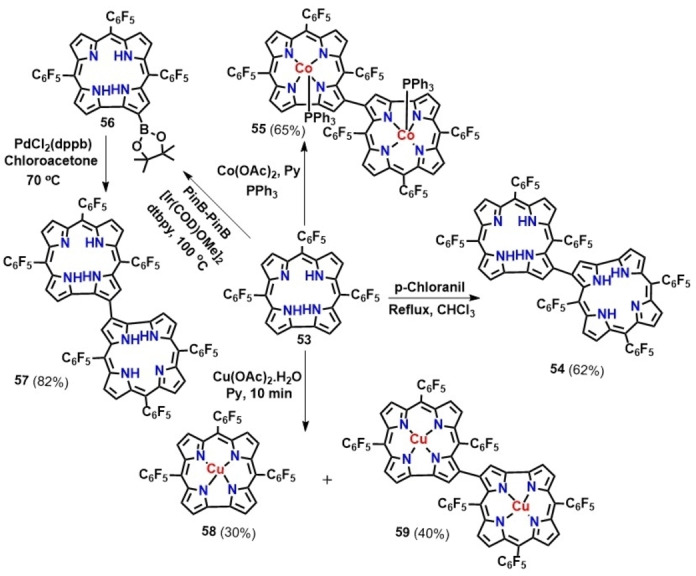
Synthetic routes for the β‐β corrole dimers.

Corroles, due to the inherent electron rich nature of the ring can easily undergo intermolecular oxidative aromatic coupling resulting in the formation of directly linked bis‐corroles. The first β‐β singly‐linked corrole dimer was synthesized by Gross and co‐workers in 2001, where they reported that addition of PPh_3_ in cobalt corroles results in the formation of 3‐3′ linked cobalt dimers (55, Scheme 6).^[51]^ Similar observation in case of copper‐containing metallocorroles (59, Scheme 6) was also reported in the literature.^[52]^ Later Osuka and coworkers reported an alternative synthetic routes for the formation of corrole dimers (54, Scheme 6) by simply refluxing corroles with p‐chloranil.^[53]^ Another report from the same group suggested the use of palladium‐catalyst for oxidative homocoupling of 2‐borylcorroles using chloroacetone as an oxidant to afford 2‐2′ linked corrole dimers (57).^[54]^

#### 3.1.2 *meso‐meso* linked

**Scheme 7:**
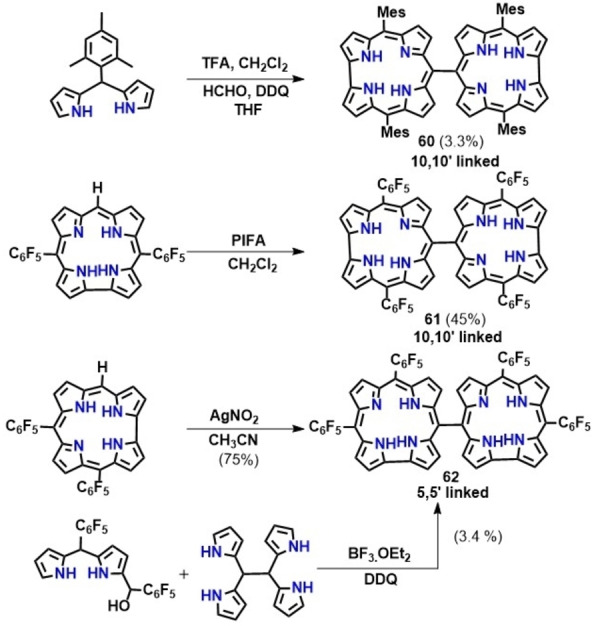
Schematic for the synthesis of *meso*‐*meso* linked corrole dimer.

*Meso‐meso* linked corrole dimer was first synthesized by Gryko and co‐workers via condensation of mesityl dipyrrane with formaldehyde in the presence of DDQ and THF as a co‐solvent resulting in the formation of 10‐10′ corrole dimers (60, Scheme 7).^[55]^ The reaction proceeds through the initial formation of 5,15‐dimesitylcorroles followed by a tandem oxidative homocoupling leading to the generation of *meso‐meso* dimers. Analogous corrole dimers (61, Scheme 7) containing four C_6_F_5_ substituents were synthesized following a similar route via oxidative homocoupling between two 5,15‐bis (pentafluorophenyl)corrole units in the presence of a strong oxidant, [Bis(trifluoroacetoxy)iodo]benzene (PIFA).^[56]^ On the other hand, using a mild oxidant such as AgNO_2_, 5‐5′‐linked corrole dimers (62, Scheme 7) were obtained in appreciable yield from the corresponding 5,10‐bis (pentafluorophenyl)corrole.^[56]^ A singly 5‐5′ linked corrole dimer could also be synthesized via condensation of a dipyrromethane‐1‐carbinol with 1,1,2,2‐tetrapyrroethane (Scheme 7).^[57]^ Electronic absorption spectrum of the meso‐meso linked corrole dimers (5‐5′, 10‐10′ linkage) displays splitting of the Soret like band due to exciton coupling between the two corroles. The UV‐Vis spectrum of complex 62 (5‐5′ linked) exhibits a moderate red‐shift of the Soret band compared to complex 61 (10‐10′), whereas Q bands of the former complex are appreciably red‐shifted relative to the analogous bands in 61.^[56]^

#### 3.1.3 Doubly linked

**Scheme 8:**
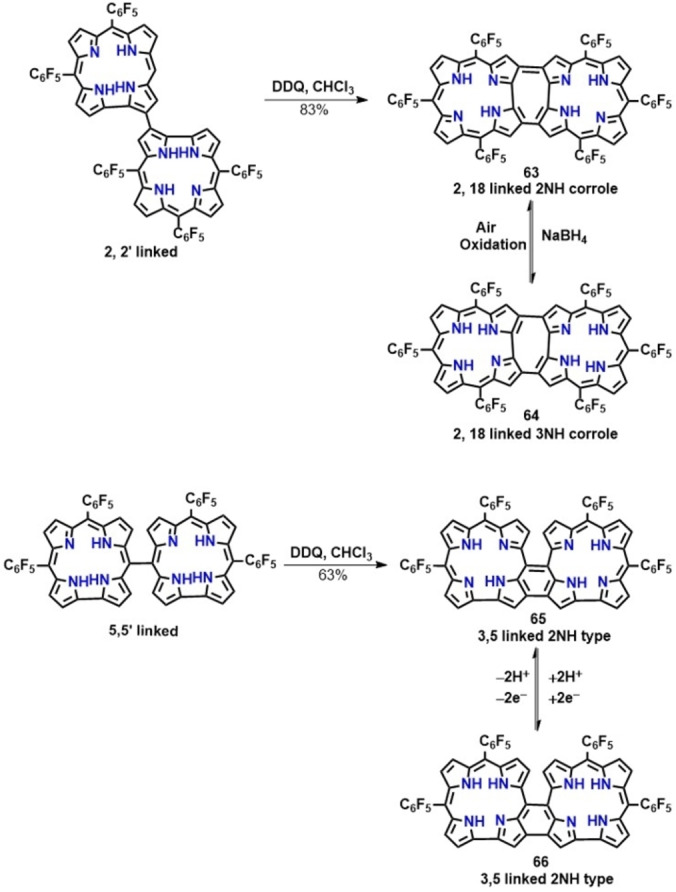
Synthetic outline for the doubly linked corrole dimer.

Synthesis of doubly 2‐18 linked corrole dimers was first reported by Osuka and co‐workers in 2006.^[54]^ They have shown that in the presence of an oxidant (DDQ), the 2‐2′ linked corrole dimer was converted into an air and moisture stable doubly‐linked 2NH‐ type corrole dimer (63, Scheme 8) which exhibited appreciable stability in spite of the singlet biradical character of the 2H‐corrole ring (Scheme 8). This corrole dimer (63, Scheme 8) exhibited a very weak absorption band around 1100–1200 nm, characteristic of antiaromatic porphyrinoids.^[54]^ This dimer so formed was further reduced by NaBH_4_ affording unstable aromatic 3NH‐type corrole dimer (64, Scheme 8) which easily reverted back to the oxidized form 63 under aerobic conditions.

The doubly 3,5‐ linked corrole dimer (65, Scheme 8) was obtained by intermolecular oxidative fusion of 5‐5′ linked corrole dimer in presence of DDQ.^[57]^ Likewise, the 2,18‐ doubly‐linked (64, Scheme 8) and the 3,5‐ doubly‐linked corrole dimers (66, Scheme 8) containing two pyrrolic NH protons upon reduction with NaBH_4_ afforded the corresponding unstable 3NH‐ type corrole dimer 66 (Scheme 8).

#### 3.1.4 Triply linked

**Scheme 9:**
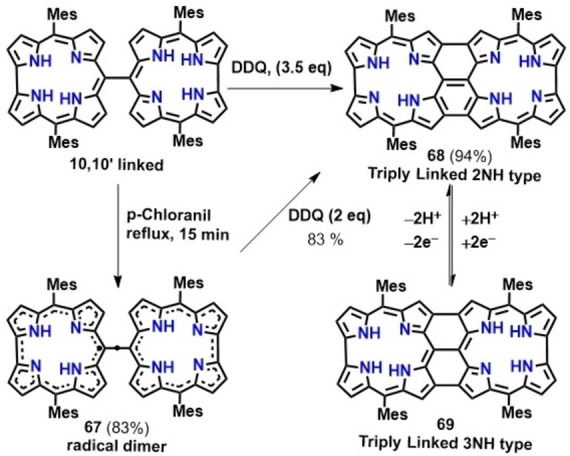
Synthesis of triply linked corrole tapes.

Triply linked 2NH‐type corrole dimer (68, Scheme 9) was synthesized via oxidation of a very dilute solution of 10‐10′ linked dimer in the presence of 3.5 equiv. of DDQ (Scheme 9).^[58]^ This compound showed characteristic ^1^H NMR and absorption spectra ascribed to non‐aromatic behavior of the corrole ring. As a consequence, fused corrole dimers display very weak absorbance profile in comparison to 10‐10′ linked dimer. Absorption spectrum of the fused corrole dimers comprises of Soret like bands at 520 nm, 546 nm as well as a broad band at 653 nm along with a weak absorption band extending well into the NIR region. This 2NH‐type corrole dimer upon reduction with NaBH_4_ converts to the corresponding aromatic 3NH‐type fused corrole (69, Scheme 9) which is relatively unstable as compared to the respective 2NH‐type corrole; therefore, it instantaneously oxidizes back to 2NH corrole dimer upon exposure to air.^[58]^ However, stable 10‐10′‐linked neutral 2NH‐type corrole radical dimer (67, Scheme 9) can be obtained by oxidation of the 10‐10′‐linked dimer with an equimolar amount of *p*‐chloranil,^[59]^ which can be further oxidized to the triply linked 2NH‐type corrole dimer (68, Scheme 9) in presence of DDQ (2 equiv.). The reaction pathway leading to the formation of stable triply‐linked corrole dimer therefore indicates that formation of triply linked corrole tapes proceeds through the involvement of a neutral stable diradical intermediate (Scheme 9).

### 3.2 Electrochemical properties

**Scheme 10:**
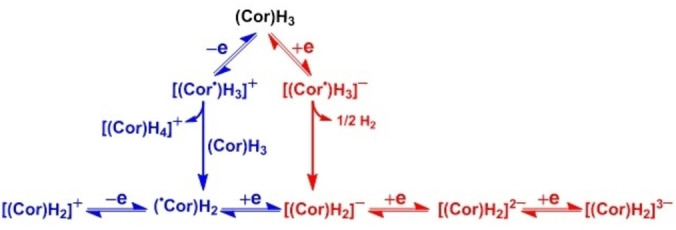
Electron transfer mechanistic pathway for [(Cor)H_3_].^[61]^

Because of its conjugated π‐framework, corrole complexes can undergo multiple oxidation and reduction depending on the nature of the electroactive metal ions, and type of axial ligands. However, unlike porphyrin, it is not always straightforward to identify the site of electron transfer in case of corroles due to the prominent non‐innocent nature of the ligand.^[60]^ For free‐base corrole, in basic solvents like pyridine or DMF, electron transfer precedes by loss of one proton, whereas in solvents like benzonitrile or DCM loss of proton occurs after one‐electron transfer. The anionic form [(Cor)H_2_]^−^, formed after the loss of one proton can further undergo successive two electron reduction leading to dianionic ([(Cor)H_2_]^2−^) and trianionic ([(Cor)H_2_]^3−^) species and two successive oxidation to give neutral (^•^Cor)H_2_ and [(Cor)H_2_]^**+**^, respectively (Scheme 10).^[61]^

Electrochemistry of metallo‐corrole monomer has been extensively studied by the groups of Kadish, Gross and Ghosh.^[62]^ Several comprehensive review articles on the electrochemistry of metallocorrole monomer are already available in the literature.^[63]^ Therefore, the discussion here will be limited to the electrochemical behavior of directly linked corrole dimers.

#### 3.2.1 β‐β singly linked

**Figure 8:**
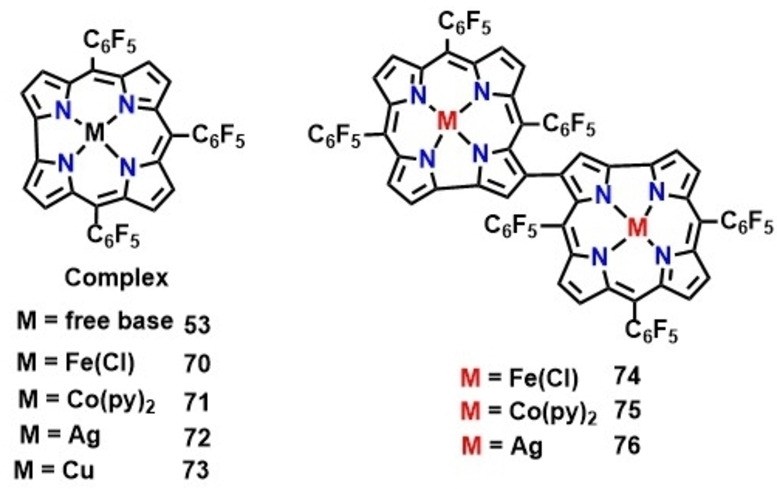
β‐β singly linked corrole dimers investigated for electrochemical studies.

**Figure 9:**
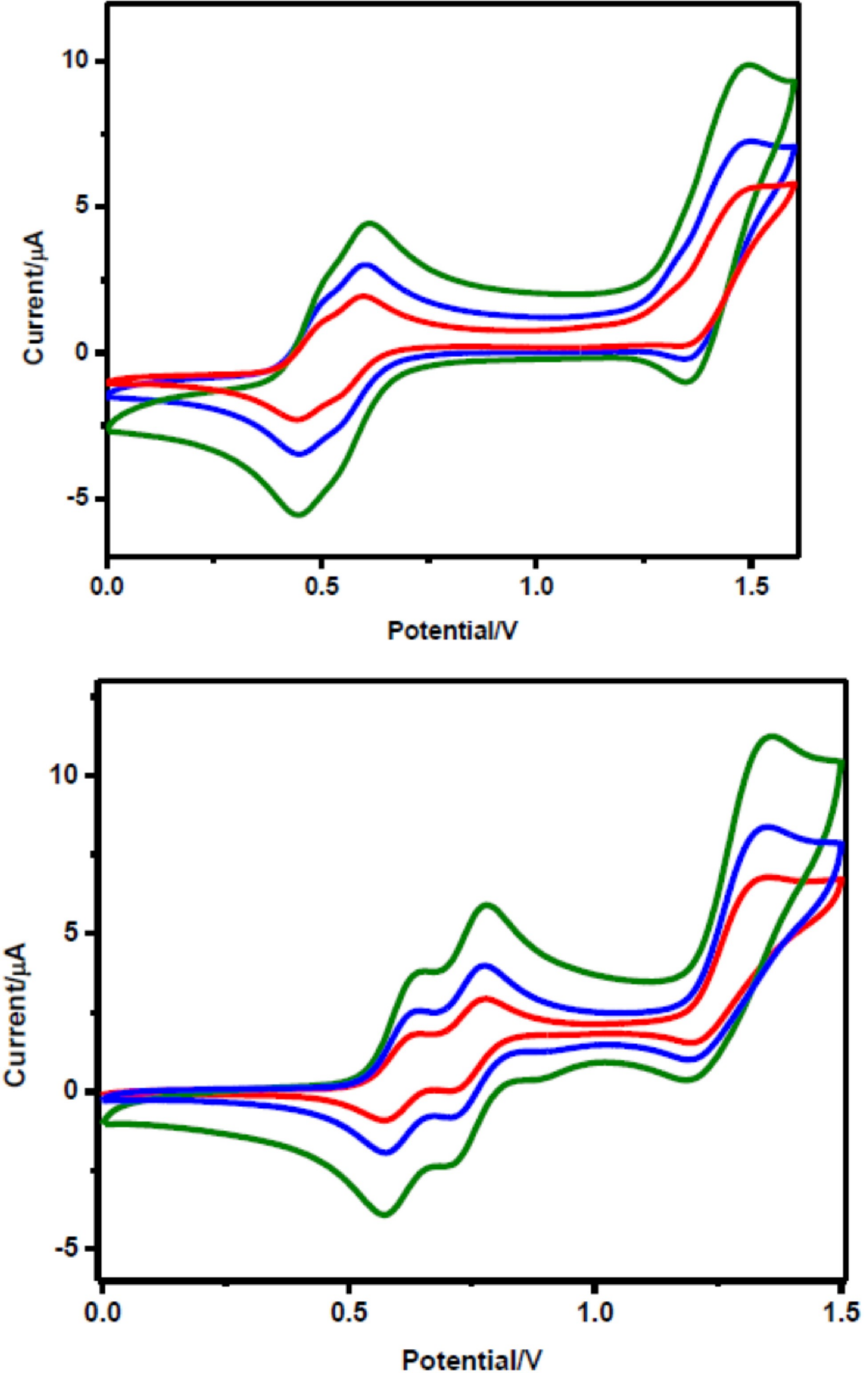
Cyclic voltammograms of 0.5 mM of **74** (top) and **75** (bottom) in acetonitrile, 0.1 M TBAP at different scan rates (0.05 V/s, 0.1 V/s, 0.25 V/s). Adapted from Ref. [64b] with permission from. ©2020 American Chemical Society.

**Figure 10:**
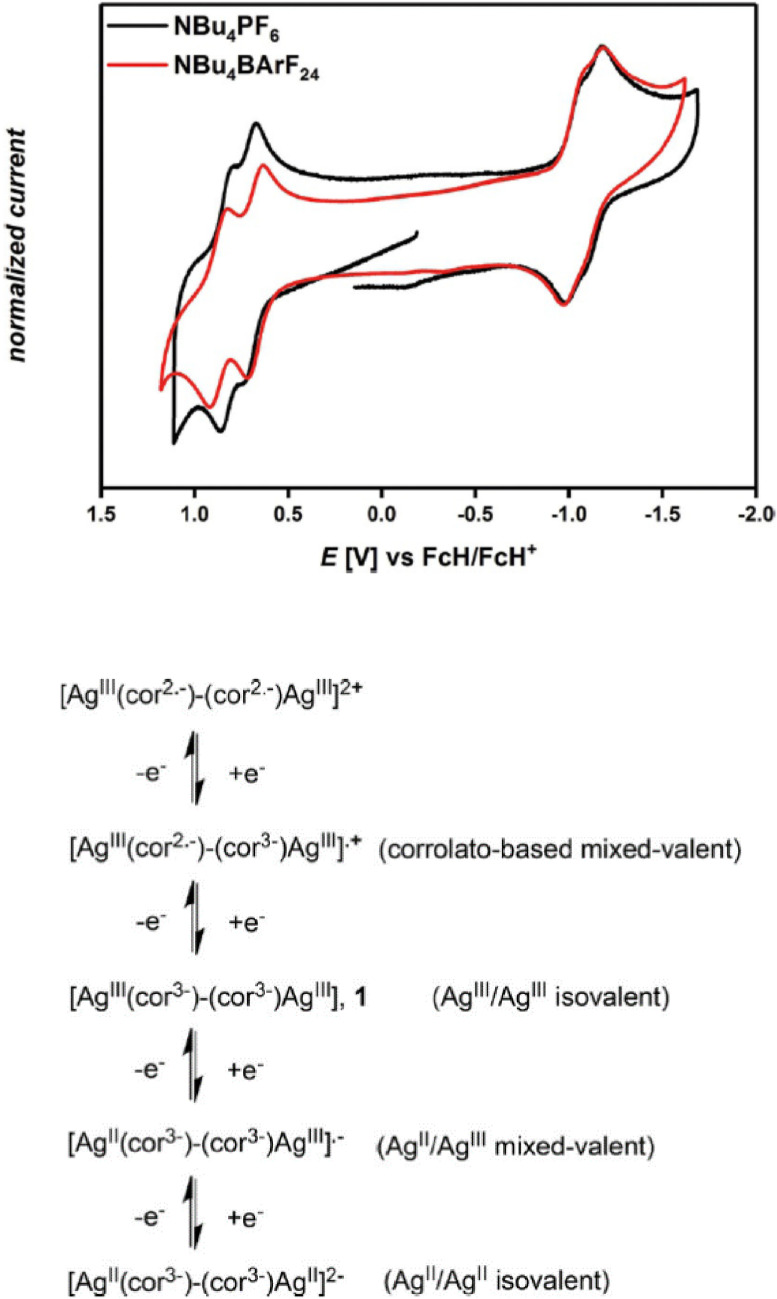
Comparison of Cyclic voltammograms of **76** measured with two different electrolytes in DCM, glassy carbon as working electrodes (left). Adapted from Ref. [64a,64c] with permission from ©2017 Wiley‐VCH GmbH.

Metal complexes of β‐β singly linked corrole dimers (**74**–**76**, Figure 8) display two closely spaced (*Δ*E_ox_∼100 mV) one electron oxidation waves as compared to single oxidation process observed in case of monomer (Figure 9).^[64]^ Poor separation between two successive oxidation processes could be attributed to weak electronic communications between two individual corrole units. Impact of the type metal ions (Fe, Co) was seen from the anodic shift in redox potential on moving from Fe‐based corrole dimers to Co‐based dimers. The third oxidation in both bimetallic corrole dimers was observed at relatively higher potential around 1.5 V. On the other hand, Ag‐containing corrole dimers also exhibit two closely spaced reduction waves in addition to oxidation, where changing the electrolyte from Bu_4_NBArF_24_ to Bu_4_NPF_6_ causes appreciable differences in half‐wave potentials of the two oxidation processes (Figure 10).

Both the β‐β linked iron and cobalt corrole dimers were further tested as electrocatalysts for water oxidation in acetonitrile along with their corresponding monomers.^[64b]^ Surprisingly, iron containing β‐β linked iron corrole dimer (74, Figure 8) displayed only a slight improvement in catalytic performances as compared to monomeric counterpart. In contrast, bimetallic cobalt corrole (75, Figure 8) exhibited superior catalytic activity ( (i_c_/i_p_)_dimer_=3×(i_c_/i_p_)_monomer_) over monomeric analogue in addition to stabilization of the catalytically active species. Investigation of redox processes via UV‐Vis‐NIR spectroelectrochemistry (SEC) and EPR‐SEC suggested metal‐centered reduction processes and ligand‐centered oxidation processes for the silver‐based corrole dimers. In case of bimetallic silver corrole, one‐electron reduction of the ground state species (Ag (III)) species resulted in the formation of rare silver‐based mixed valent form [Ag^II^(cor^3−^)‐(cor^3−^)Ag^III^]^•‐^ whereas, the one‐electron oxidized species could be represented as corrolato based mixed‐ valent species (Figure 10).[^64a^, ^64c^]

#### 3.2.2 *meso‐meso* linked

**Figure 11:**
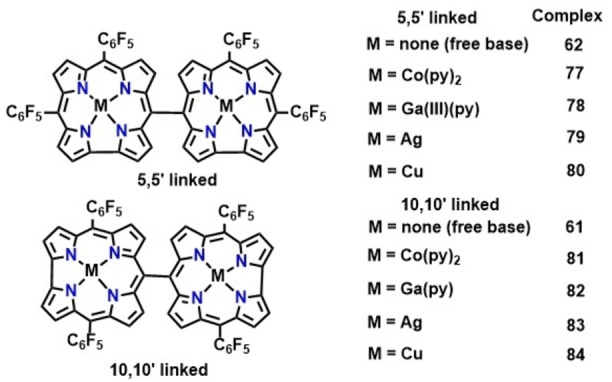
Meso‐meso singly linked corrole dimers investigated for electrochemical studies.

The position of the *meso*‐*meso* linkage (5,5′ or 10,10′) has a significant impact on the electrochemical behaviour of *meso*‐*meso* linked bimetallic corrole dimers. Osuka and co‐workers investigated electrochemical properties of different metal (Co, Ag, Cu and Ga, 77–80 and 81–84, Figure 11 and Table 3) complexes of *meso*‐*meso* linked corrole dimers.^[65]^ They reported that metal complexes of both monomeric and dimeric species exhibit one/two oxidation and several closely spaced reduction processes along with an irreversible first reduction wave. The first reduction feature could be assigned to metal‐based process, Co(III)/Co(II) as demonstrated by Gross and co‐workers using Co‐containing mononuclear corrole (71, Figure 8), in which irreversible nature of the redox wave originates from the dissociation of pyridine upon reduction.^[66]^

**Table 3:** Electrochemical potentials of metallocorrole monomer and meso‐meso linked bis metallocorroles.[^65^, ^66^]

Complexes	Oxidation		Reduction			
	$E_{ox2}^{a}$	$E_{ox1}^{a}$	$E_{red1}^{a}$	$E_{red2}^{\;a}$	$E_{red3}^{\;a}$	$E_{red4}^{\;a}$
**62**	0.33	0.13^[b,c]^	−1.51^[c]^	−1.71^[c]^	–	–
**77**	0.17	−0.04	−0.82^[c]^	−2.01^[b]^	−2.16^[b,c]^	−2.30^[b,c]^
**78**	0.35^[b,c]^	0.13^[b,c]^	−2.01^[c]^	−2.23^[b,c]^	–	–
**79**	0.75^[c]^	0.52	−1.06	−1.19^[b,c]^	−2.07^[c]^	–
**80**	0.60^[b]^	0.46^[b,c]^	−0.30	−0.48	−2.39^[b,c]^	–
**61**	0.49^[b,c]^	0.27^[b,c]^	−1.64^[c]^	2.39^[c]^	–	–
**81**	0.19	−0.02	−0.97^[c]^	−2.00^[b]^	−2.17^[b,c]^	‐2.29^[b,c]^
**82**	0.28	0.12	−1.93^[c]^	−2.37^[c]^	–	–
**83**	0.70	0.54	−1.06	−1.20^[b,c]^	−2.08^c^	–
**84**	0.61	0.50^[b,c]^	−0.32^[c]^	−0.51	−2.42^[c]^	–
**53**	0.52^[c]^	0.42^[c]^	−1.51^[c]^	−2.12	–	–
**71**	–	0.15	−0.72^[b,c]^	−1.93		
**72**	–	0.67	−1.07	−1.99^[c]^	2.14	–
**73**	–	0.62	−0.28	−2.20^[b,c]^	−2.32^[b,c]^	–

[a] Potential determined vs. FcH/FcH^+^ in benzonitrile, working electrode: glassy carbon, counter electrode: Pt wire, Reference electrode: Ag/AgClO_4,_ scan rate: 0.05 V/s; [b] determined by differential pulse voltammetry; [c] Irreversible peaks.

**Scheme 11:**
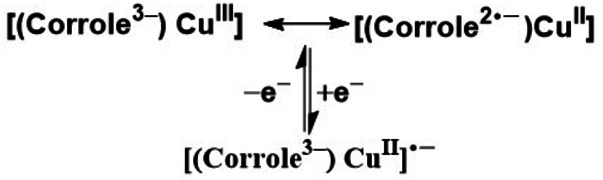
Assignment of oxidation state.

Cyclic voltammograms of the dicobalt complexes of *meso*‐*meso* linked (5,5′ or 10,10′ linkages) corroles displayed cathodic shift of both the reduction and oxidation processes over the monomeric analogue (71, Figure 8), ascribed to lack of electron withdrawing functional groups per corrole unit. While, the shift in potential in 5,5′‐linked corrole dimer (77, Figure 11) was relatively minimal, significantly large potential shift in the case of 10,10′‐linked corrole dimer (81, Figure 11) was observed, which can be explained from preferable electronic communication through the 10‐10′ linkage. A similar trend was displayed by gallium and silver complexes (79 and 83, Figure 11) of *meso*‐*meso* linked corrole dimers.^[66b]^ A study by Kar, Sarkar and co‐workers on Ag‐containing corrole dimers revealed metal‐ and ligand‐based reduction and oxidation processes respectively as also observed in case of the β‐β linked silver‐corrole dimer (76, Figure 10).[^64a^, ^64c^] On the contrary, redox processes in the respective Cu‐containing corrole dimers could be assigned to metal‐based oxidation and ligand‐based reduction processes. The assignment was based on the analysis provided by Kar, Sarkar and co‐workers in case of Cu‐containing corrole monomer, where they suggested the ground state neutral species to be a resonance hybrid of a Cu(III) corrole and a Cu(II) cation radical (Scheme 11).^[67]^

#### 3.2.3 Doubly linked

**Figure 12:**
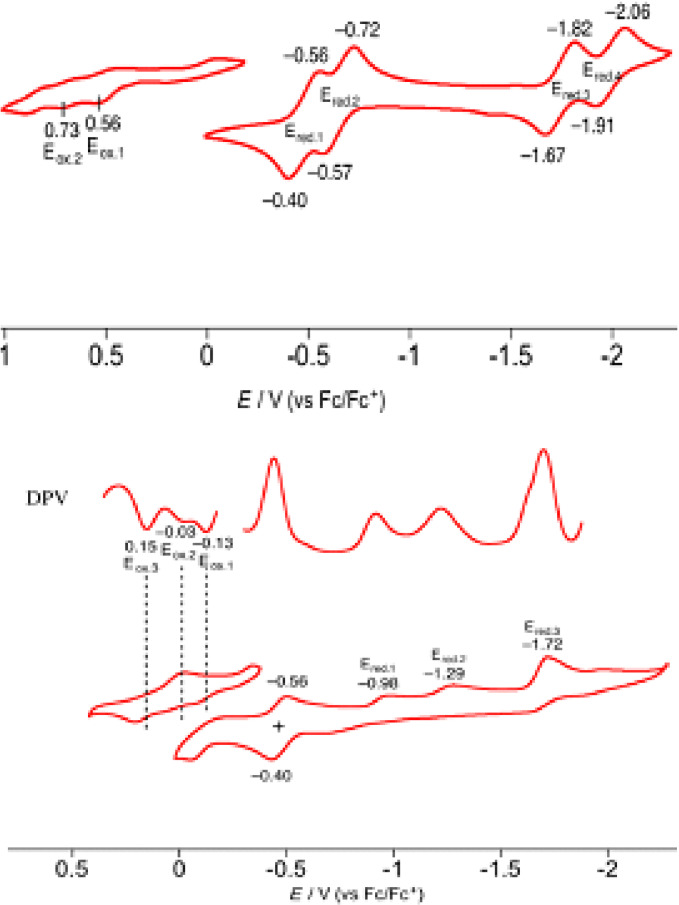
Cyclic voltammograms of **65** and **66** in benzonitrile. Working electrode: glassy carbon. Counter electrode: Pt wire. Reference electrode: Ag/AgClO_4_. Scan rate: 0.05 V/s. Adapted from ref. ^[57]^ with permission from ©2015 Wiley‐VCH GmbH.

In contrast to rather poorly separated redox waves in case of free base, and singly‐linked (*meso ‐meso* and β‐β linked) metallocorrole dimers, the doubly linked free base corrole dimers (65 and 66, scheme 8 and Figure 12) exhibit well‐defined and separated oxidation and reduction processes (Figure 12) reflecting improved electronic coupling between the individual corrole units.^[57]^ Comparison of cyclic voltammograms of both the complexes revealed significantly lower oxidation potential (−0.13 V) of 3NH‐type corrole dimer (66, Scheme 8) in comparison with complex 65. Such a low oxidation potential could be cited as a reason for rapid oxidation of 66 to 65 upon exposure to air.

#### 3.2.4 Triply linked

**Figure 13:**
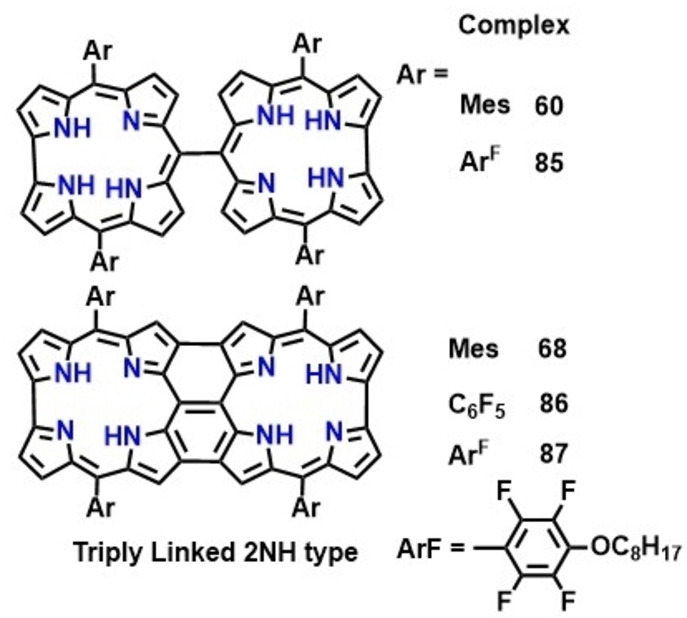
Meso‐meso and fused corrole dimers investigated for electrochemical studies.

Fused corroles display multiple redox features ascribed to extensive delocalization across the entire π ‐framework of the ligand facilitating smooth transfer of electrons across the metal‐ligand interface. Besides, effective electronic communications between two corrole units also cause significant lowering of the energy gap between HOMO and LUMO, which is responsible for appreciable lowering in redox potentials of fused corroles as compared to *meso*‐*meso* linked derivatives. Cyclic voltammograms of free base fused corrole (68, Table 4 and Figure 13) comprise of reversible, well‐defined two oxidation and reduction waves as compared to irreversible redox processes of respective *meso*‐*meso* linked free base corrole (60, Table 4 and Figure 13). Additionally, electronic properties (electron withdrawing or donating) of the functional groups present at the peripheral positions induces significant modulation of electrochemical HOMO‐LUMO gap leading to a shift (cathodic or anodic) in oxidation and reduction potentials. Therefore, redox potentials of free base corroles containing electron withdrawing groups (86, 87, Table 4 and Figure 13) were anodically shifted as compared to respective corrole dimers having electron‐donating functional groups (68, Table 4 and Figure 13).

**Table 4:** Electrochemical potentials of metal complexes of meso‐meso linked and fused bis‐corroles.[^58^, ^68^]

Complexes	Oxidation			Reduction		
	$E_{ox2}$	$E_{ox1}$	$E_{red1}$	$E_{red2}$	$E_{red3}$	ΔE [eV]
**60**	0.34	0.00	−1.81^[a]^	−1.91^[a]^		1.81
**85**	0.48^[a]^	0.16^[a,b]^	−1.72^[a]^	−2.25^[a]^		1.88
**67**	0.44	0.31	−0.52	−0.74		
**68**	0.57	0.30	−0.79	−1.07	−2.20^[a]^	1.09
**68Zn** ^[c]^	0.24	−0.13	−0.97	−1.39		0.84
**68Cu**	0.46	0.07	−0.62	−1.03	−2.18	0.69
**86**	0.68	0.43	−0.55	−0.75	−1.83	0.98
**87**	0.69	0.42	−0.59	−0.80	−1.89	1.01
**87Zn** ^[c]^	0.32^[b]^	−0.02	−0.73	−1.11		0.71

[a] irreversible peak; [b] determined by DPV, ΔE(eV)= HOMO‐LUMO gap; [c] in presence of one drop of pyridine, measured in benzonitrile. Working electrode: glassy carbon, Counter electrode: Pt wire. Reference electrode: Ag/AgClO4. Scan rate: 0.05 V/s.

**Figure 14:**
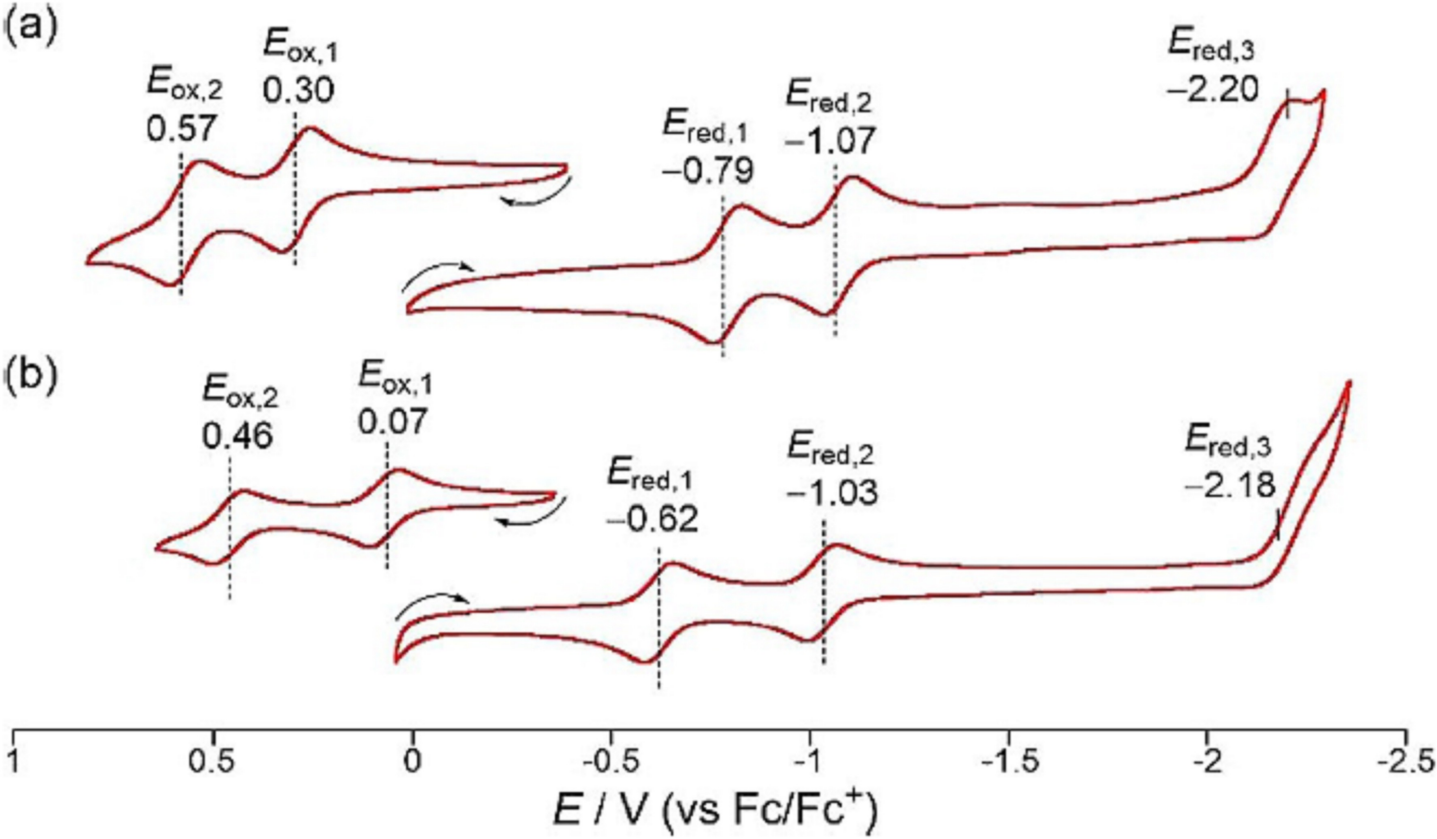
Cyclic voltammograms of **68** and **68Cu** in benzonitrile. Working electrode: glassy carbon. Counter electrode: Pt wire. Reference electrode: Ag/AgClO_4_. Scan rate: 0.05 V/s. Adapted from Ref. [68] with permission from ©2019 Wiley‐VCH GmbH.

Following a similar trend as free base fused corroles, metal complexes of these corroles also showed two reversible oxidation and reduction processes, however the value of the oxidation potential was found to be slightly lower in the latter (Figure 14).

Electrochemical properties of directly linked corrole dimers, due to the electron rich core of the ligand, provide a suitable cavity for the stabilization of high valent oxidation states of metals. Furthermore, judicious choices of metal, axial ligand and peripheral functional groups allows rational fine tuning of electrochemical potential which can be exploited in electrochemical small molecule activation reactions.

### 3.3 Magnetic Properties

**Figure 15:**
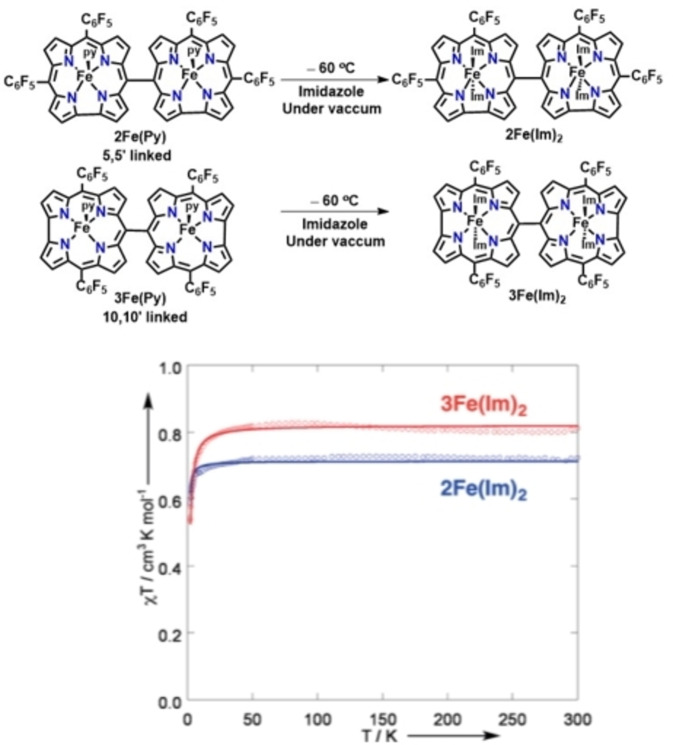
Temperature‐dependent magnetic susceptibility plots of **2Fe(Im)_2_** (blue) and **3Fe(Im)_2_** (red) measured on microcrystalline samples by using SQUID magnetometry (dot: observed, line: simulated). Reproduced from Ref. [69] with permission from ©2017 Wiley‐VCH GmbH.

The analysis of type and extent of interactions between metal center sitting at the cavity of directly linked (*meso*‐*meso* or fused) corrole dimers require measurements of temperature dependent magnetic susceptibility. Osuka and co‐workers investigated magnetic properties of Fe‐ containing *meso*‐*meso* and triply fused corrole dimers in the presence of weak (pyridine) and strong field (imidazole) axial ligands to draw a comparison between the extent of communication between the two metal centers in these two types of corrole dimers.[^68^, ^69^] They demonstrated that the *meso*‐*meso* linked Fe(III)‐corrole in the presence of a weak axial ligand like pyridine could be designated as five‐coordinate, intermediate spin (*S*=3/2) species, while strong field ligand like imidazole stabilized six‐coordinate low spin (*S*=1/2) moiety.^[70]^ To explore the impact of position of linkage (5,5′ or 10,10′) on spin‐spin magnetic interactions between two metal centers, EPR spectroscopy in combination with magnetic measurements were utilized. EPR spectroscopic measurements of both Fe(III)‐containing 5,5′‐ and10,10′‐ linked *meso‐meso* dimers (2Fe(Im)_2_ and 3Fe(Im)_2_ Figure 15) displayed rhombic spectra with a corresponding *g*‐value of 2.05 and 2.23, suggesting two isolated low spin Fe(III) corroles and two magnetically interacting Fe (III) corroles in bimetallic 5,5′ and 10,10′‐ linked corrole dimers respectively.^[69]^ The temperature‐dependent magnetic susceptibility (*χ*T) measurement revealed that the magnetic susceptibility (*χ*T) value decreased below 20 K for both the 5,5′‐ and 10,10′‐ linked *meso‐meso* dimers due to metal‐metal exchange interactions (*J*), whereas *χ*T value for monomeric iron(III) corrole coordinated with imidazole remained temperature‐independent in the range of 2–330 K. Due to larger antiferromagnetic interaction between two iron (III) centres in the 10‐10′ linked corrole dimer (3Fe(Im)_2_, Figure 15), larger exchange coupling (*J*) value of −2.04 cm^−1^ as compared to *J*=−0.64 cm^−1^ in case of 5,5′‐dimer (2Fe(Im)_2_, Figure 15) was observed.

**Figure 16:**
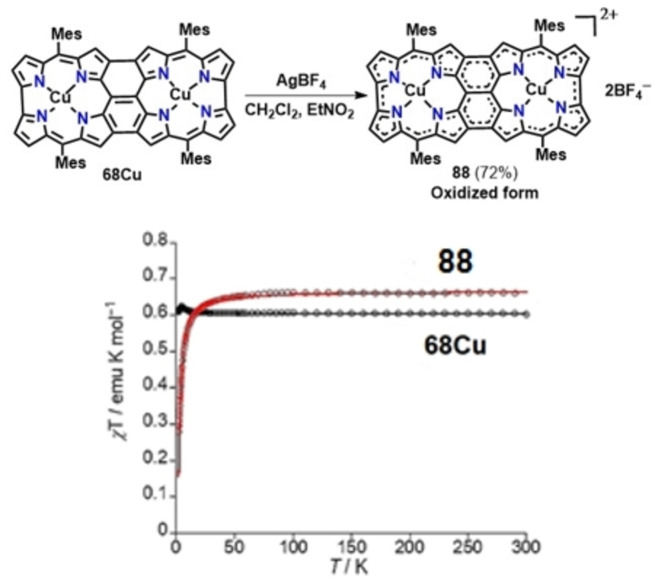
**(a)** Oxidation of triply linked copper corrole **68Cu**; **(b)** Temperature‐dependent magnetic susceptibility plots of **68Cu** (black) and **88**(red) measured at 0.5 T (dot: observed, line: simulated) (right side). Reproduced from Ref. [68] with permission from ©2019 Wiley‐VCH GmbH.

Triply linked corrole dimer (68Cu, Figure 16), on the other hand exhibited weak ferromagnetic interaction with *J*=+0.90 cm^−1^(+1.30 K) whereas the oxidized form (88, Figure 16) showed antiferromagnetic interaction between two Cu^II^ centers with *J*=‐1.83 cm^−1^(−2.63 K) due to larger spin densities value at the (C10, C10′) positions (92, Figure 16) than that of parent species (68Cu, Figure 16).^[68]^ In comparison with triply linked corrole dimer 68Cu, the triply linked porphyrin dimer 48 exhibited antiferromagnetic interaction with *J* value of −1.43 cm^−1^(−2.06 K),^[22a]^ attributed to the difference in spin‐transporting pathways based on the length of C10, C10′ linkages. A combined experimental and theoretical analysis suggested that longer and shorter C10, C10′ bond lengths in triply fused porphyrin and corrole dimers facilitate spin transport through β‐β and *meso*‐*meso* bonds respectively, giving rise to weak intramolecular spin‐interactions in the corrole containing dimer.

## 4 Conclusion

Rich coordination chemistry of metal complexes of bis‐porphyrinoids (porphyrins or corroles) combined with a wide range of intriguing physicochemical properties have already highlighted the benefits of covalently linked ligands as compared to respective monomeric porphyrins. Significant improvement in the synthetic strategies in recent years resulted in sizeable number of bimetallic complexes of bis‐porphyrinoids being reported in the literature. Usually, metal complexes of multiple linked fused (triply‐ or doubly‐ fused) complexes exhibit clearly distinct physicochemical properties as compared to corresponding singly‐linked compounds, attributed to the efficient delocalization of π‐electron cloud across the entire ligand backbone due to the coplanar arrangement of the porphyrin units. In contrast, singly‐linked metalloporphyrinoids due to orthogonal disposition of porphyrin subunits enable the limited interporphyrin electronic communication resulting in similarity in electrochemical and optical properties with the monomer. From the perspective of coordination chemistry, directly linked porphyrin and corrole dimers can essentially accommodate two different metals in varied oxidation states allowing investigation of magnetic interactions and engineer binuclear catalytic centers, where multimetallic centers can act in a concerted way towards activation of a substrate. Moreover, higher activity of these dimers towards electrocatalytic small molecule activation indeed suggest use of these extended macrocycle as a design element for designing robust catalysts. Additionally, remarkable photophysical properties of metal complexes of these frameworks such as the extremely red‐shifted NIR absorption bands and excellent conductivity make them suitable candidates for potential applications in non‐linear optical materials (NLO), NIR sensors and dyes, and molecular‐scale electronic devices. However, despite significant advancements in the chemistry of covalently linked metalloporphyrinoids in the last decades or so, proportionate growth in the coordination chemistry of directly linked bisporphyrinoids has not been observed due to various synthetic shortcomings like low yield, aggregation, and solubility issues. Therefore, careful tuning of various factors like electronic properties of functional groups or axially coordinated ligands, redox non‐innocence of metal centers is necessary for a better understanding of the coordination chemistry of these class of macrocyclic compounds. Since metal complexes of directly linked bis‐porphyrins already demonstrated outstanding applications across wide range of research disciplines, further analysis related to the impact of extended conjugation on the activity of these complex warrant further analysis. Moreover, fundamental investigations of the superior activity of the bimetallic porphyrinoids are particulary interesting because it will likely open new avenues for developing novel functional materials. In this context, various interesting attributes related to the physicochemical properties of the directly linked bis‐porphyrins highlighted in this review are expected to stimulate further investigation of these type of structural motif as a promising candidate for numerous applications across wide range of research disciplines.

## Conflict of interest

The authors declare no conflict of interest.

5

## Biographical Information

*Arijit Singha Hazari obtained his Ph.D. degree from the Indian Institute of Technology (IIT), Bombay, in 2019 under the supervision of Prof. G. K. Lahiri in the direction of electronic structural aspects of metal complexes of selective non‐innnocent ligands and their potential applications in homogeneous catalysis. He then moved to Prof. Sarkar's group at University of Stuttgart as a Marie‐Curie postdoctoral research fellow. His current research interests are primarily centered around designing transition metal complexes of fused porphyrionoids and investigation of their electrocatalytic applications*.

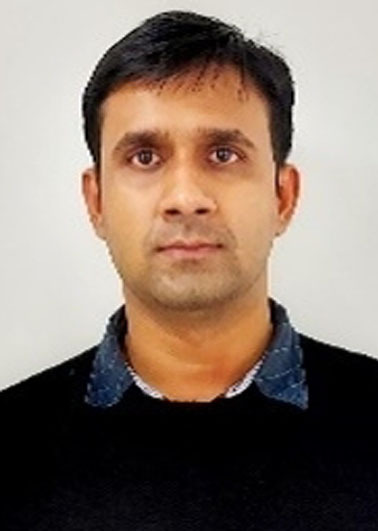

## Biographical Information

*Shubhadeep Chandra received his MSc degree from Indian Institute of Technology Bombay, India. In 2021, he obtained his Ph.D. from University of Stuttgart, Germany under the supervision of Prof. Biprajit Sarkar. His doctoral research focused on synthesis of extended porphyrin derivatives and investigation of their electrocatalytic activities. Currently, he is working in Prof. Schuhmann's group at Ruhr‐University Bochum as a postdoctoral research associate and his area of research interest is synthesis of redox polymers for applications in bioelectrochemistry*.

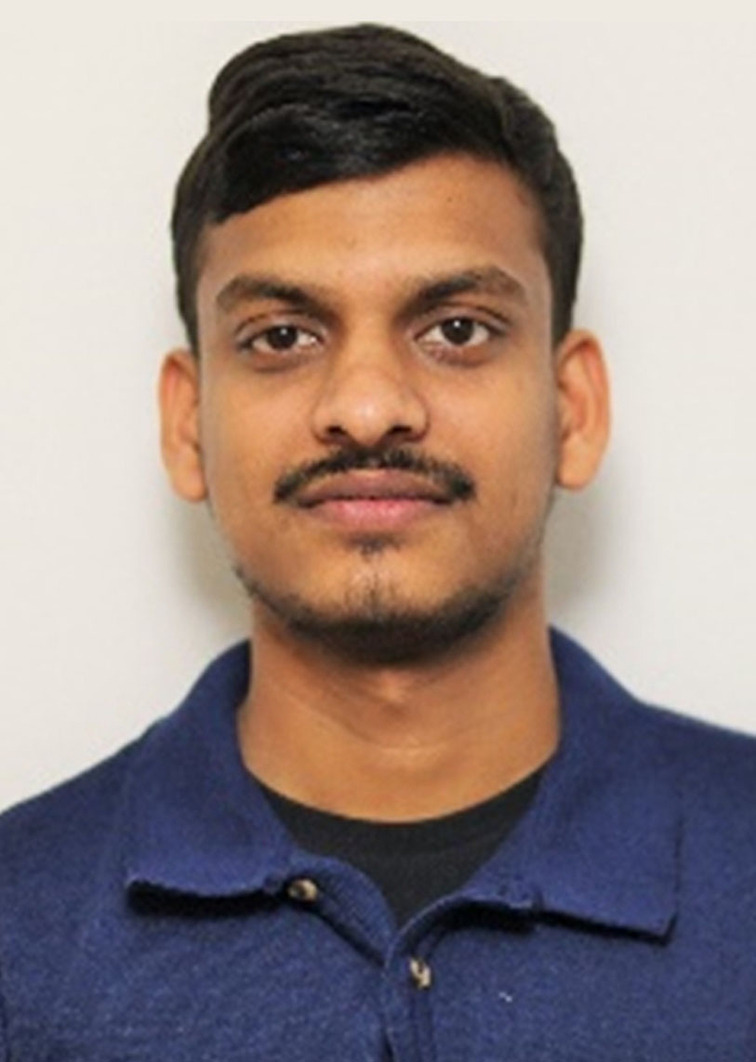

## Biographical Information

*Sanjib Kar studied chemistry at The University of Burdwan and the University of Kalyani (West Bengal), India, and received his Ph.D. from the IIT Bombay, India, in December 2005 under the supervision of Prof. G. K. Lahiri. In April 2006, he moved to Kyushu University, Japan, as a JSPS postdoctoral fellow to work with Prof. Yoshinori Naruta. He joined the NISER‐Bhubaneswar as an assistant professor in 2009. Since 2016, he has been an Associate Professor at the School of Chemical Sciences, NISER Bhubaneswar. His research focuses on the synthesis and application of porphyrinoid and metalloporphyrinoid‐based molecules*.

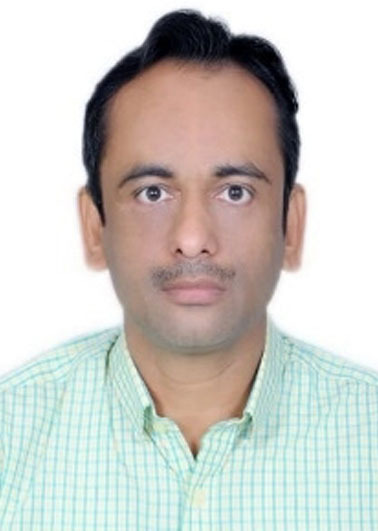

## Biographical Information

*Biprajit Sarkar studied chemistry at the University of Delhi and at IIT Bombay. In 2005 he completed his doctoral work under the supervision of Prof. W. Kaim at the University of Stuttgart. Between 2005 and 2006 Biprajit was a post‐doctoral researcher in the group of Prof. P. Braunstein in Strasbourg. He worked on his habilitation between 2006 and 2011. From 2012 until 2019 he was a W2 Professor at the FU Berlin. Since October 2019 Biprajit is a W3 Professor and chair of inorganic coordination chemistry at the University of Stuttgart. His research interests are in the fields of redox‐active ligands, mesoionic carbenes and porphyrinoid systems, with a strong focus on the investigation of the physical properties of metal complexes of these ligands, and their use in (electro)catalysis, in optically and magnetically switchable systems, and in medicinal chemistry*.

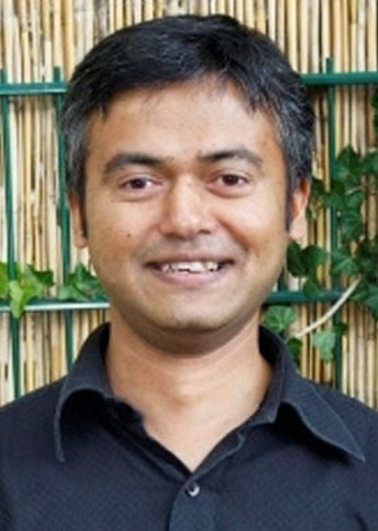

## Data Availability

Data sharing is not applicable to this article as no new data were created or analyzed in this study.
